# Supplementary material for: Connecting moss lipid droplets to patchoulol biosynthesis
Source: PLoS One. 2020 Dec 7;15(12):e0243620. doi: 10.1371/journal.pone.0243620 (PMC7721168; doi:10.1371/journal.pone.0243620)
Supplement: S1 File — (PDF) [file pone.0243620.s001.pdf]

Supporting information

Includes engineering strategy, primer list, GC-MS data, qPCR data, gene expression data including expression shown in the moss development stages from the eFP browser, LD size distribution and LD and Seipin localization

Construct name	Expressed protein	Protein location
PpOle1	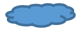	ER/LD
PpOle1-Venus	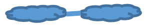	ER/LD
PpOle1-PTS	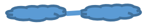	ER/LD
PpOle1-LP4/2A-PTS	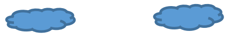	ER/LD/Cytosol
PpSeipin325	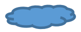	ER
PpSeipin325-Venus	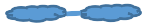	ER
PpSeipin325-PTS	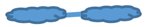	ER
AtLDAP1	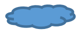	ER/LD
AtLDAP1-Venus	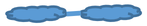	ER/LD
PpLDAP1-PTS	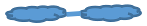	ER/LD
PTS	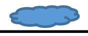	Cytosol

S1 Table. Gene names of constructs designed for this experiment. The protein nature of the expressed proteins with or without a linker. The predicted localization of the proteins.

S2 Table: (F: Forward primer, R: Reverse primer).

Primers for gene overexpression

Primer name		Sequence 5'-3'
pRH 4.7 kb	F	CCAGATCGACCACATCCTTCTCCG
pRH 4.7 kb	R	GACCTGCAGAAGTAACACCAAACAACAG
pRH 2.1 kb	F	GTCCTGCTTTAATGAGATATGCGAGACG
pRH 2.1 kb	R	ACGAAGGCCGTTCTTCCCTG
PpOLE	F	CTGTTGTTTGGTGTTACTTCTGCAGGTCATGGATAATGCCAAAACCAAGGC
PpOLE	R	CGTCTCGCATATCTCATTAAAGCAGGACAGCCGCGACGCTGGTATC
PpSeipin	F	CTGTTGTTTGGTGTTACTTCTGCAGGTCATGGCTTCCTCCGACGTC
PpSeipin	R	CGTCTCGCATATCTCATTAAAGCAGGACCTAGTTTTGATCCAGAACCTTTCC
AtLDAP1	F	CTGTTGTTTGGTGTTACTTCTGCAGGTCATGGAGACAGAGAAGAAAAA
AtLDAP1	R	CGTCTCGCATATCTCATTAAAGCAGGACCTACTCCGAATCAGACGATG
PTS	F	CTGTTGTTTGGTGTTACTTCTGCAGGTCATGGAGTTGTATGCCCAAAGT
PTS	R	CGTCTCGCATATCTCATTAAAGCAGGACTTAATATGGAACAGGGTGAAGG

Primers for localization studies

Primer name		Sequence 5'-3'
pRH 4.7 kb	F	CCAGATCGACCACATCCTTCTCCG
pRH 4.7 kb	R	GACCTGCAGAAGTAACACCAAACAACAG
pRH 2.1 kb + Venus	F	GGTAGCGGCAGCGGTAGCGGTAGCGGCAGCATGGTGAGCAAGGGCGAGG
pRH 2.1 kb	R	ACGAAGGCCGTTCTTCCCTG
PpOLE	F	CTGTTGTTTGGTGTTACTTCTGCAGGTCATGGATAATGCCAAAACCAAGGC
PpOLE	R	GCTGCCGCTACCGCTACCGCTGCCGCTACCAGCCGCGACGCTGGTATC
PpSeipin	F	CTGTTGTTTGGTGTTACTTCTGCAGGTCATGGCTTCCTCCGACGTC
PpSeipin	R	TGTAGCCACTTCATCTGCAGCATTAGAGTTTTGATCCAGAACCTTTCC
AtLDAP1	F	CTGTTGTTTGGTGTTACTTCTGCAGGTCATGGAGACAGAGAAGAAAAA
AtLDAP1	R	GCTGCCGCTACCGCTACCGCTGCCGCTACCCTCCGAATCAGACGATG

Primers for LD attached PTS lines

Primer name		Sequence 5'-3'
pRH 4.7 kb	F	CCAGATCGACCACATCCTTCTCCG
pRH 4.7 kb	R	GACCTGCAGAAGTAACACCAAACAACAG
pRH 2.1 kb	F	GTCCTGCTTTAATGAGATATGCGAGACG
pRH 2.1 kb	R	ACGAAGGCCGTTCTTCCCTG
PTS	F	GGTAGCGGCAGCGGTAGCGGTAGCGGCAGCATGGAGTTGTATGCCCAAAGT
PTS	R	CGTCTCGCATATCTCATTAAAGCAGGACTTAATATGGAACAGGGTGAAGG
PpOLE	F	CTGTTGTTTGGTGTTACTTCTGCAGGTCATGGATAATGCCAAAACCAAGGC
PpOLE	R	GCTGCCGCTACCGCTACCGCTGCCGCTACCAGCCGCGACGCTGGTATC
PpSeipin	F	CTGTTGTTTGGTGTTACTTCTGCAGGTCATGGCTTCCTCCGACGTC
PpSeipin	R	TGTAGCCACTTCATCTGCAGCATTAGAGTTTTGATCCAGAACCTTTCC
AtLDAP1	F	CTGTTGTTTGGTGTTACTTCTGCAGGTCATGGAGACAGAGAAGAAAAA
AtLDAP1	R	GCTGCCGCTACCGCTACCGCTGCCGCTACCCTCCGAATCAGACGATG

Primers for genotyping and qPCR

Primer name		Sequence 5'-3'
Genotype	F	CCTGCCTTCATACGCTATTTATTTGCT
Genotype	R	CAACGTGCACAACAGAATTGAAAGC
PTS	F	TGTCTTGGAGTGGAAGAGG
PTS	R	GTGTCCTGTAATATCATTGACGA
Actin	F	GGACTTCGAGCAGGAGTT
Actin	R	TCATTATCGAGTTGTACGTCG
β-tubulin	R	GAGCCATACAATGCTACCC
β-tubulin	R	AGGAGTAATGAGTTTCAGTGTCC

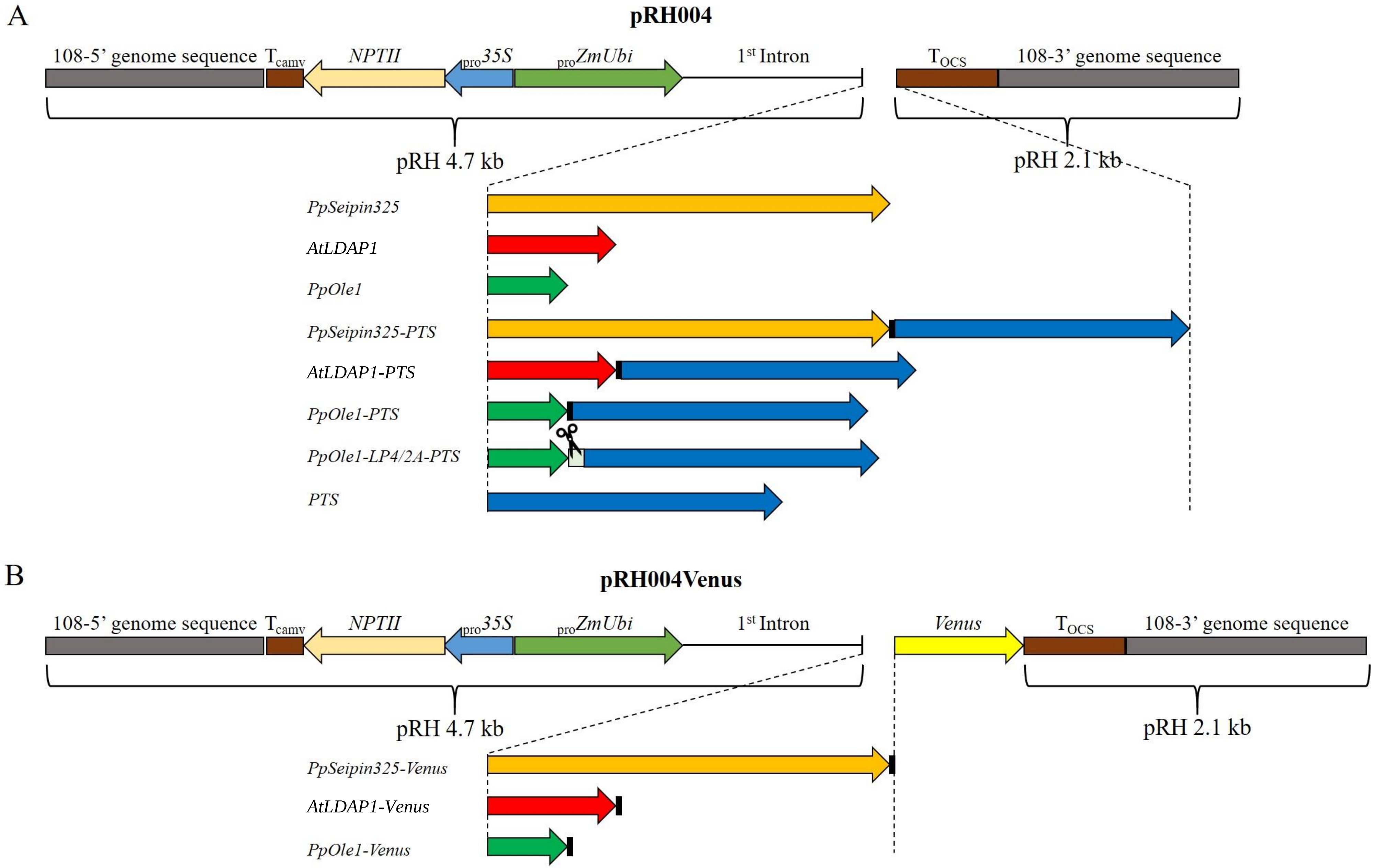

S1 Fig. Construction of cell lines. A): The pRH 4.7 kb region and pRH 2.1 kb region were amplified from the pRH004 plasmid, and transformed with the necessary fragments for overexpression studies. B): For the localization studies, the pRH004 plasmid was built with Venus as shown and transformed with appropriate PCR amplified fragments. All fragments were gel purified.

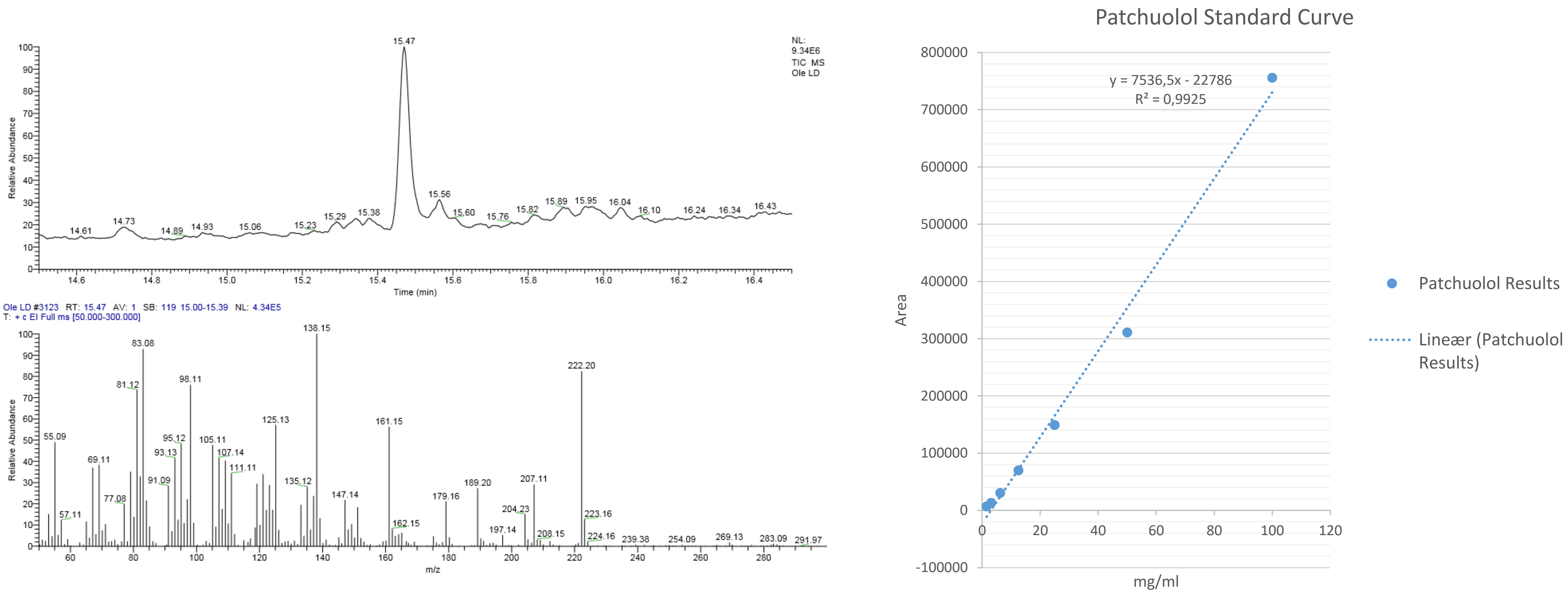

S2 Fig. Qualitative and Quantitative GC-MS analysis of patchoulol concentrated in the LDs of *P. patens* lines. Left hand side is from the *PpOle1-PTS* line. Standard curve for Patchoulol from GC-MS analysis

.

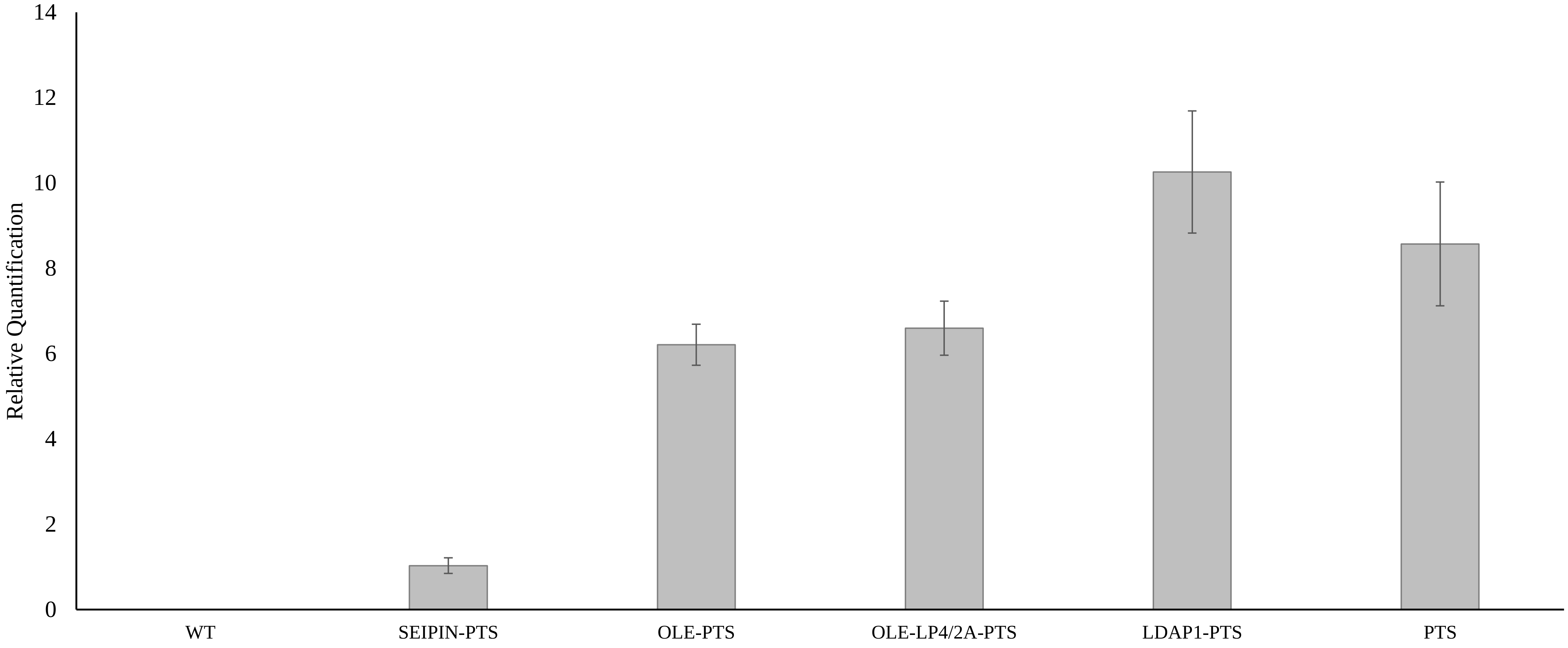

S3 Fig. qPCR quantification of PTS in each line. The expression of PTS in the *ZmUbi:PTS* line is about 8.5 times higher than the *ZmUbi:PpSeipin325-PTS* line. This can explain some of the differences observed in the PTS levels. The standard deviation is shown as bars in the figure.

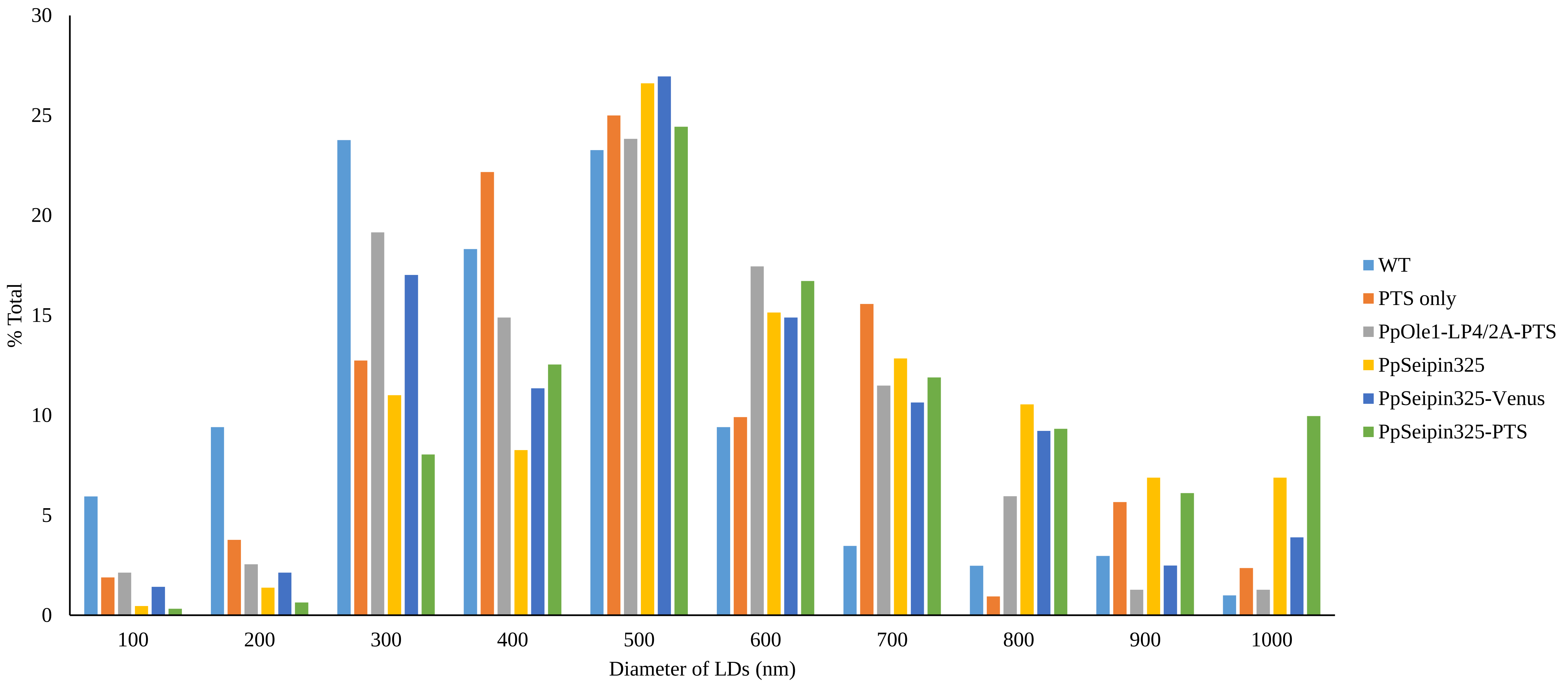

S4 Fig. Percentage distribution of the LDs in different sizes (diameter in nm) for each of *P. patens* line. This figure show that Seipin expression provides larger LDs and Oleosin.

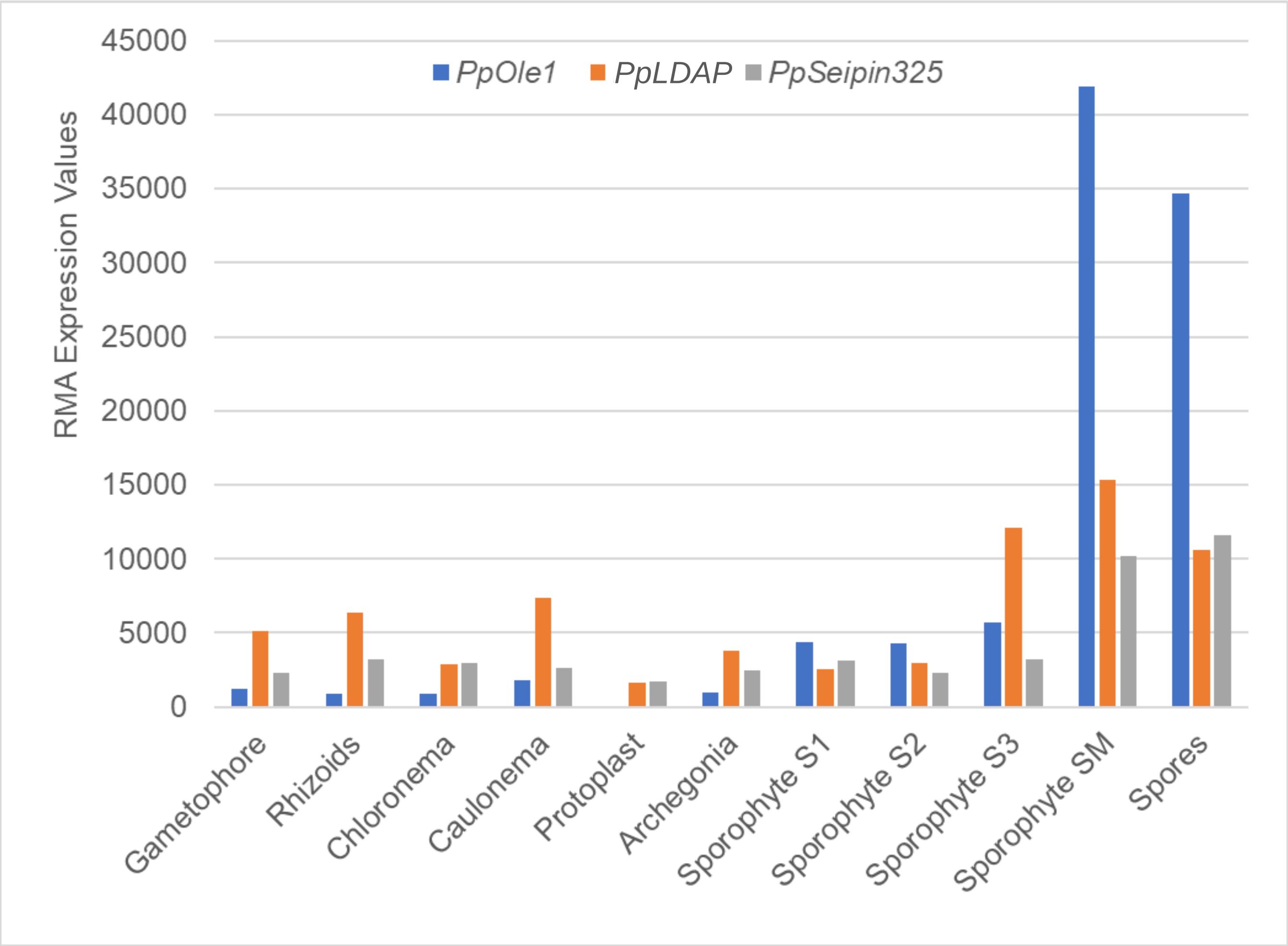

S5 Fig. Tissue- or developmental stage-specific gene expression level of three lipid droplet-associated protein genes; *PpOle1*, *PpLDAP* and *PpSeipin325*.

The gene expression data was obtained from *Physcomitrella* eFP Browser (http://bar.utoronto.ca/efp_physcomitrella/cgi-bin/efpWeb.cgi) to analyze the gene expression of three lipid body protein genes in different tissue or different developmental stage. All three genes showed high level of expression specifically in mature sporophyte (SM) and spores. Expression data were normalized by Robust Multi-array Average (RMA). It show, that the protonemal tissue that normally occur in bioreactors do not have high expression of the three studies genes.

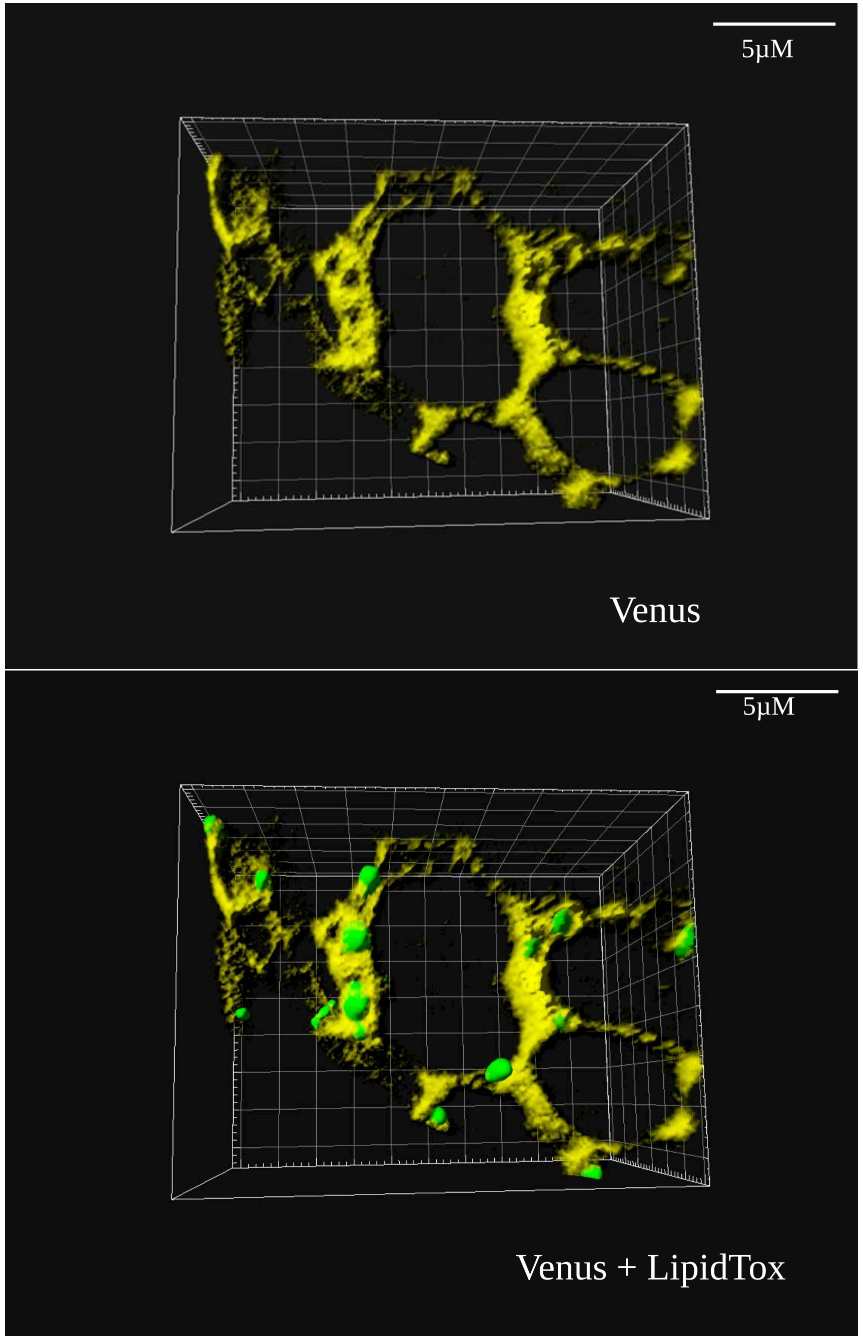

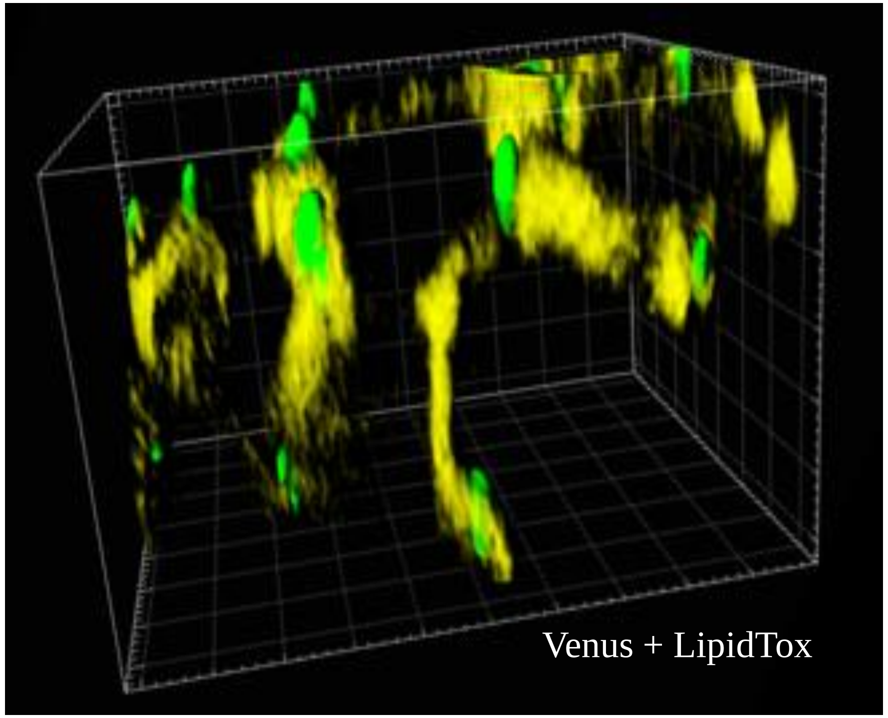

S6 Fig. Different angles of the image shown in figure 4B.
This 3D image shows the LDs in green and the Venus protein localization of Seipin325 from a different angle.
