# Supplementary material for: Connecting moss lipid droplets to patchoulol biosynthesis
Source: PLoS One. 2020 Dec 7;15(12):e0243620. doi: 10.1371/journal.pone.0243620 (PMC7721168; doi:10.1371/journal.pone.0243620)
Supplement: S2 File — Genes 256-fold differentially expressed between the mutants are shown in tables of the individual mutants. (PDF) [file pone.0243620.s002.pdf]

Differential expressed genes in Lipid bodies.

Genes 256 fold differentially expressed between the mutants.

	**325**	**Ole-LP4-PTS**	**Ole-PTS**	**PTS**	**LDAP-PTS**	**WT**
325	0	0	1	0	0	65
Ole-LP4-PTS	0	0	9	8	0	58
Ole-PTS	1	9	0	1	9	160
PTS	0	8	1	0	10	214
LDAP-PTS	0	0	9	10	0	17
WT	65	58	160	214	17	0

The list below is for the individual mutants, with the highest differences towards WT
PpSeipin325 is TRINITY_DN33985_c0_g2_i6
For the mutant 325 the top 25 list includes (yellow is part of the integration plasmid):

	325_1	Ole-LP4-PTS_1	Ole-PTS_1	Ole-PTS_2	PTS_1	PTS_2	LDAP-PTS_1	WT_1	WT_2
**TRINITY_DN38215_c0_g1**	1.122.518	0,099	0,116	0,154	0,07	0,03	0	19.948	3.228
**TRINITY_DN50091_c6_g1**	895.130	487.929	780.646	646.413	1.094.400	1.062.918	693.377	2.808	1.765
**TRINITY_DN5532_c0_g1**	516.614	212.740	340.040	298.596	501.545	497.402	292.483	0,848	0,797
**TRINITY_DN49887_c0_g1**	407.396	409.522	364.535	345.914	429.405	420.770	226.078	0,455	0,494
**TRINITY_DN17926_c0_g1**	354.116	412.054	361.551	317.894	310.472	319.082	20.319	0,313	0,091
**TRINITY_DN38568_c0_g1**	308.059	318.474	318.204	283.525	409.788	419.345	205.826	0,677	0,474
**TRINITY_DN50002_c0_g1**	242.095	0,01	0,021	0,019	0	0	0	3.707	0,565
**TRINITY_DN50465_c0_g1**	227.983	103.004	177.539	144.260	220.215	223.451	179.259	0,919	0,605
**TRINITY_DN44118_c21_g1**	171.545	497.621	2.988.097	2.589.358	2.583.480	2.661.563	5.737.899	0,263	0,424
**TRINITY_DN929_c0_g1**	143.373	129.993	111.031	107.181	139.801	135.391	75.454	0,222	0,081
**TRINITY_DN32998_c0_g1**	134.013	87.582	99.367	104.213	138.281	141.987	60.785	0,212	0,171
**TRINITY_DN11934_c0_g1**	128.166	44.605	80.074	74.416	126.998	123.885	63.216	0,313	0,504
**TRINITY_DN34029_c1_g1**	112.605	637.773	2.189.608	1.961.225	537.918	541.147	1.549.930	1.323	0,504
**TRINITY_DN32681_c2_g1**	108.301	77.126	93.735	76.088	143.615	141.120	67.175	0,424	0
**TRINITY_DN38263_c0_g2**	106.268	68.279	90.908	84.137	110.351	111.212	41.710	0,242	0,464
**TRINITY_DN61444_c0_g1**	98.253	57.157	60.129	51.650	98.273	96.418	25.284	0,131	0
**TRINITY_DN24061_c0_g1**	96.773	63.175	75.713	67.884	89.969	90.281	54.965	0,131	0,161
**TRINITY_DN29463_c0_g1**	96.346	0	0	0	0	0	0	1.111	0,272
**TRINITY_DN32215_c0_g1**	95.022	69.967	79.254	63.245	98.750	96.966	62.665	0,121	0,121
**TRINITY_DN32215_c0_g2**	68.518	51.676	51.502	46.742	75.428	77.280	45.688	0,121	0
**TRINITY_DN6081_c0_g1**	67.778	60.205	59.898	59.047	85.261	84.672	48.822	0,091	0,091
**TRINITY_DN6494_c0_g1**	66.006	42.212	43.946	44.408	59.715	59.805	29.984	0,03	0,05
**TRINITY_DN55842_c0_g1**	64.985	102.100	62.336	60.075	71.991	74.699	72.283	0,162	0,04
**TRINITY_DN2561_c0_g1**	60.492	69.579	65.467	61.083	68.147	67.427	53.313	0,111	0
**TRINITY_DN34803_c0_g1**	53.395	18.301	43.673	31.661	51.997	51.616	35.747	0,051	0,212

With the following annotation (from integration)

**Contig in sequencing assembly**	P. patens NCBI #	**Description**
**TRINITY_DN38215_c0_g1**		Ribulose bisphosphate carboxylase large chain {ECO:0000255|HAMAP-Rule:MF_01338}
**TRINITY_DN50091_c6_g1**	PNR39672	hypothetical protein PHYPA_019951 [Physcomitrella patens]
**TRINITY_DN5532_c0_g1**	XP_024379832 or XP_024380033	uncharacterized protein LOC112284343 [Physcomitrella patens] or alpha carbonic anhydrase 7-like [Physcomitrella patens]
**TRINITY_DN49887_c0_g1**	XP_024393312	aldehyde oxidase GLOX-like [Physcomitrella patens]
**TRINITY_DN17926_c0_g1**	XP_024365953 or XP_024368032	uncharacterized protein LOC112277638 isoform X2 [Physcomitrella patens] or alpha carbonic anhydrase 5-like [Physcomitrella patens]
**TRINITY_DN38568_c0_g1**	XP_024393797	uncharacterized protein LOC112291067 [Physcomitrella patens]
**TRINITY_DN50002_c0_g1**	NP_904175.1	PSII 47kDa protein (chloroplast) [Physcomitrella patens]
**TRINITY_DN50465_c0_g1**	XP_024363543	non-specific lipid-transfer protein 3-like [Physcomitrella patens]
**TRINITY_DN44118_c21_g1**	ACK58633	kanamycin resistance protein [Cloning vector pHELLSGATE]
**TRINITY_DN929_c0_g1**	XP_024395254	chalcone synthase 6-4-like [Physcomitrella patens] or ABU87504 2'-oxoalkylresorcinol synthase [Physcomitrella patens]
**TRINITY_DN32998_c0_g1**	XP_024363339	abscisic acid 8'-hydroxylase 3-like [Physcomitrella patens]
**TRINITY_DN11934_c0_g1**	XP_024368003	uncharacterized protein LOC112278633 [Physcomitrella patens]
**TRINITY_DN34029_c1_g1**	ALW83197	YFP-CcmM35 fusion protein (chloroplast) [synthetic construct]
**TRINITY_DN32681_c2_g1**	PNR53501	hypothetical protein PHYPA_007176 [Physcomitrella patens]
**TRINITY_DN38263_c0_g2**	XP_024381396	uncharacterized protein LOC112285094 [Physcomitrella patens]
**TRINITY_DN61444_c0_g1**	XP_024388184	pathogen-related protein-like [Physcomitrella patens]

**TRINITY_DN24061_c0_g1**	XP_024368984	GDSL esterase/lipase At4g16230-like [Physcomitrella patens]
**TRINITY_DN29463_c0_g1**	NP_904206.1	PSII 44 kD protein (chloroplast) [Physcomitrella patens]
**TRINITY_DN32215_c0_g1**	PNR26297	hypothetical protein PHYPA_030871 [Physcomitrella patens] / UDP-glycosyltransferase 83A1 (quercetin 3/7-O-glucosyltransferase activity)
**TRINITY_DN32215_c0_g2**	PNR26297	hypothetical protein PHYPA_030871 [Physcomitrella patens] / UDP-glycosyltransferase 83A1
**TRINITY_DN6081_c0_g1**	XP_024395980	peroxidase 21-like isoform X1 [Physcomitrella patens] or XP_024395981 peroxidase 21-like isoform X2 [Physcomitrella patens]
**TRINITY_DN6494_c0_g1**	XP_024382012	GDSL esterase/lipase At5g03820-like [Physcomitrella patens]
**TRINITY_DN55842_c0_g1**	XP_024377855	probable xyloglucan endotransglucosylase/hydrolase [Physcomitrella patens]
**TRINITY_DN2561_c0_g1**	XP_024385936	probable pectinesterase 15 [Physcomitrella patens]
**TRINITY_DN34803_c0_g1**	XP_024369838	uncharacterized protein LOC112279537 [Physcomitrella patens]<br>stigma-specific STIG1-like protein 1 [Helianthus annuus]

For the mutant PpOle-LP4-PTS_1 the top 25 list includes (yellow is part of the integration plasmid):

Ole-LP4-PTS_1 constitute the following contigs:

	325_1	Ole-LP4-PTS_1	Ole-PTS_1	Ole-PTS_2	PTS_1	PTS_2	LDAP-PTS_1	WT_1	WT_2
**TRINITY_DN34029_c1_g1**	112.605	637.773	2.189.608	1.961.225	537.918	541.147	1.549.930	1.323	0,504
**TRINITY_DN44118_c21_g1**	171.545	497.621	2.988.097	2.589.358	2.583.480	2.661.563	5.737.899	0,263	0,424
**TRINITY_DN50091_c6_g1**	895.130	487.929	780.646	646.413	1.094.400	1.062.918	693.377	2.808	1.765
**TRINITY_DN17926_c0_g1**	354.116	412.054	361.551	317.894	310.472	319.082	20.319	0,313	0,091
**TRINITY_DN49887_c0_g1**	407.396	409.522	364.535	345.914	429.405	420.770	226.078	0,455	0,494
**TRINITY_DN38568_c0_g1**	308.059	318.474	318.204	283.525	409.788	419.345	205.826	0,677	0,474
**TRINITY_DN5532_c0_g1**	516.614	212.740	340.040	298.596	501.545	497.402	292.483	0,848	0,797
**TRINITY_DN929_c0_g1**	143.373	129.993	111.031	107.181	139.801	135.391	75.454	0,222	0,081
**TRINITY_DN50465_c0_g1**	227.983	103.004	177.539	144.260	220.215	223.451	179.259	0,919	0,605
**TRINITY_DN55842_c0_g1**	64.985	102.100	62.336	60.075	71.991	74.699	72.283	0,162	0,04
**TRINITY_DN32629_c33_g1**	10.923	96.142	1.187	3.314	12.863	7.890	0	2.505	0,827
**TRINITY_DN32998_c0_g1**	134.013	87.582	99.367	104.213	138.281	141.987	60.785	0,212	0,171
**TRINITY_DN55646_c0_g1**	51.831	80.274	26.082	22.660	59.138	61.798	54.908	0,141	0,071
**TRINITY_DN32681_c2_g1**	108.301	77.126	93.735	76.088	143.615	141.120	67.175	0,424	0
**TRINITY_DN38832_c0_g1**	53.207	70.423	40.195	40.767	57.659	54.037	30.307	0,061	0,252
**TRINITY_DN32215_c0_g1**	95.022	69.967	79.254	63.245	98.750	96.966	62.665	0,121	0,121
**TRINITY_DN2561_c0_g1**	60.492	69.579	65.467	61.083	68.147	67.427	53.313	0,111	0
**TRINITY_DN38263_c0_g2**	106.268	68.279	90.908	84.137	110.351	111.212	41.710	0,242	0,464
**TRINITY_DN24061_c0_g1**	96.773	63.175	75.713	67.884	89.969	90.281	54.965	0,131	0,161
**TRINITY_DN6081_c0_g1**	67.778	60.205	59.898	59.047	85.261	84.672	48.822	0,091	0,091
**TRINITY_DN61444_c0_g1**	98.253	57.157	60.129	51.650	98.273	96.418	25.284	0,131	0
**TRINITY_DN16400_c0_g1**	46.974	53.374	29.781	30.364	42.293	44.005	39.175	0,111	0,111
**TRINITY_DN32215_c0_g2**	68.518	51.676	51.502	46.742	75.428	77.280	45.688	0,121	0
**TRINITY_DN11934_c0_g1**	128.166	44.605	80.074	74.416	126.998	123.885	63.216	0,313	0,504
**TRINITY_DN6494_c0_g1**	66.006	42.212	43.946	44.408	59.715	59.805	29.984	0,03	0,05

**Contig in sequencing assembly**	P. patens NCBI #	**Description**
**TRINITY_DN34029_c1_g1**	ALW83197	YFP-CcmM35 fusion protein (chloroplast) [synthetic construct]
**TRINITY_DN44118_c21_g1**	ACK58633	kanamycin resistance protein [Cloning vector pHELLSGATE]
**TRINITY_DN50091_c6_g1**	PNR39672	hypothetical protein PHYPA_019951 [Physcomitrella patens]
**TRINITY_DN17926_c0_g1**	XP_024365953 or XP_024368032	uncharacterized protein LOC112277638 isoform X2 [Physcomitrella patens] or alpha carbonic anhydrase 5-like [Physcomitrella patens]
**TRINITY_DN49887_c0_g1**	XP_024393312	aldehyde oxidase GLOX-like [Physcomitrella patens]
**TRINITY_DN38568_c0_g1**	XP_024393797	uncharacterized protein LOC112291067 [Physcomitrella patens]
**TRINITY_DN5532_c0_g1**	XP_024379832 or XP_024380033	uncharacterized protein LOC112284343 [Physcomitrella patens] or alpha carbonic anhydrase 7-like [Physcomitrella patens]
**TRINITY_DN929_c0_g1**	XP_024395254	chalcone synthase 6-4-like [Physcomitrella patens] or ABU87504 2'-oxoalkylresorcinol synthase [Physcomitrella patens]
**TRINITY_DN50465_c0_g1**	XP_024363543	non-specific lipid-transfer protein 3-like [Physcomitrella patens]
**TRINITY_DN55842_c0_g1**	XP_024377855	probable xyloglucan endotransglucosylase/hydrolase [Physcomitrella patens]
**TRINITY_DN32629_c33_g1**		unknown
**TRINITY_DN32998_c0_g1**	XP_024363339	abscisic acid 8'-hydroxylase 3-like [Physcomitrella patens]
**TRINITY_DN55646_c0_g1**	XP_024375379	non-specific lipid-transfer protein 2G-like [Physcomitrella patens]
**TRINITY_DN32681_c2_g1**	PNR53501	hypothetical protein PHYPA_007176 [Physcomitrella patens]
**TRINITY_DN38832_c0_g1**	PNR53501	hypothetical protein PHYPA_007176 [Physcomitrella patens]
**TRINITY_DN32215_c0_g1**	PNR26297	hypothetical protein PHYPA_030871 [Physcomitrella patens] / UDP-glycosyltransferase 83A1 (quercetin 3/7-O-glucosyltransferase activity)
**TRINITY_DN2561_c0_g1**	XP_024385936	probable pectinesterase 15 [Physcomitrella patens]
**TRINITY_DN38263_c0_g2**	XP_024381396	uncharacterized protein LOC112285094 [Physcomitrella patens]

**TRINITY_DN24061_c0_g1**	XP_024368984	GDSL esterase/lipase At4g16230-like [Physcomitrella patens]
**TRINITY_DN6081_c0_g1**	XP_024395980	peroxidase 21-like isoform X1 [Physcomitrella patens] or XP_024395981 peroxidase 21-like isoform X2 [Physcomitrella patens]
**TRINITY_DN61444_c0_g1**	XP_024388184	pathogen-related protein-like [Physcomitrella patens]
**TRINITY_DN16400_c0_g1**	XP_024395390	receptor-like protein 51 [Physcomitrella patens]
**TRINITY_DN32215_c0_g2**	PNR26297	hypothetical protein PHYPA_030871 [Physcomitrella patens] / UDP-glycosyltransferase 83A1 (quercetin 3/7-O-glucosyltransferase activity)
**TRINITY_DN11934_c0_g1**	XP_024368003	uncharacterized protein LOC112278633 [Physcomitrella patens]
**TRINITY_DN6494_c0_g1**	XP_024382012	GDSL esterase/lipase At5g03820-like [Physcomitrella patens]

For the mutant PpOle-PTS_1+2 the top 25 list includes (yellow is part of the integration plasmid):

Ole-PTS_1+2 constitute the following contigs:

	325_1	Ole-LP4-PTS_1	Ole-PTS_1	Ole-PTS_2	PTS_1	PTS_2	LDAP-PTS_1	WT_1	WT_2
**TRINITY_DN44118_c21_g1**	171.545	497.621	2.988.097	2.589.358	2.583.480	2.661.563	5.737.899	0,263	0,424
**TRINITY_DN34029_c1_g1**	112.605	637.773	2.189.608	1.961.225	537.918	541.147	1.549.930	1.323	0,504
**TRINITY_DN50091_c6_g1**	895.130	487.929	780.646	646.413	1.094.400	1.062.918	693.377	2.808	1.765
**TRINITY_DN49887_c0_g1**	407.396	409.522	364.535	345.914	429.405	420.770	226.078	0,455	0,494
**TRINITY_DN17926_c0_g1**	354.116	412.054	361.551	317.894	310.472	319.082	20.319	0,313	0,091
**TRINITY_DN5532_c0_g1**	516.614	212.740	340.040	298.596	501.545	497.402	292.483	0,848	0,797
**TRINITY_DN38568_c0_g1**	308.059	318.474	318.204	283.525	409.788	419.345	205.826	0,677	0,474
**TRINITY_DN50465_c0_g1**	227.983	103.004	177.539	144.260	220.215	223.451	179.259	0,919	0,605
**TRINITY_DN929_c0_g1**	143.373	129.993	111.031	107.181	139.801	135.391	75.454	0,222	0,081
**TRINITY_DN32998_c0_g1**	134.013	87.582	99.367	104.213	138.281	141.987	60.785	0,212	0,171
**TRINITY_DN32681_c2_g1**	108.301	77.126	93.735	76.088	143.615	141.120	67.175	0,424	0
**TRINITY_DN38263_c0_g2**	106.268	68.279	90.908	84.137	110.351	111.212	41.710	0,242	0,464
**TRINITY_DN11934_c0_g1**	128.166	44.605	80.074	74.416	126.998	123.885	63.216	0,313	0,504
**TRINITY_DN32215_c0_g1**	95.022	69.967	79.254	63.245	98.750	96.966	62.665	0,121	0,121
**TRINITY_DN24061_c0_g1**	96.773	63.175	75.713	67.884	89.969	90.281	54.965	0,131	0,161
**TRINITY_DN2561_c0_g1**	60.492	69.579	65.467	61.083	68.147	67.427	53.313	0,111	0
**TRINITY_DN55842_c0_g1**	64.985	102.100	62.336	60.075	71.991	74.699	72.283	0,162	0,04
**TRINITY_DN61444_c0_g1**	98.253	57.157	60.129	51.650	98.273	96.418	25.284	0,131	0
**TRINITY_DN6081_c0_g1**	67.778	60.205	59.898	59.047	85.261	84.672	48.822	0,091	0,091
**TRINITY_DN32215_c0_g2**	68.518	51.676	51.502	46.742	75.428	77.280	45.688	0,121	0
**TRINITY_DN6494_c0_g1**	66.006	42.212	43.946	44.408	59.715	59.805	29.984	0,03	0,05
**TRINITY_DN34803_c0_g1**	53.395	18.301	43.673	31.661	51.997	51.616	35.747	0,051	0,212
**TRINITY_DN38832_c0_g1**	53.207	70.423	40.195	40.767	57.659	54.037	30.307	0,061	0,252
**TRINITY_DN61594_c0_g1**	43.525	22.710	35.256	30.479	46.306	46.724	15.191	0,202	0,101
**TRINITY_DN26375_c0_g1**	36.083	27.158	33.911	31.843	40.783	42.739	39.004	0	0

**Contig in sequencing assembly**	P. patens NCBI #	**Description**
**TRINITY_DN44118_c21_g1**	ACK58633	kanamycin resistance protein [Cloning vector pHELLSGATE]
**TRINITY_DN34029_c1_g1**	ALW83197	YFP-CcmM35 fusion protein (chloroplast) [synthetic construct]
**TRINITY_DN50091_c6_g1**	PNR39672	hypothetical protein PHYPA_019951 [Physcomitrella patens]
**TRINITY_DN49887_c0_g1**	XP_024393312	aldehyde oxidase GLOX-like [Physcomitrella patens]
**TRINITY_DN17926_c0_g1**	XP_024365953 or XP_024368032	uncharacterized protein LOC112277638 isoform X2 [Physcomitrella patens] or alpha carbonic anhydrase 5-like [Physcomitrella patens]
**TRINITY_DN5532_c0_g1**	XP_024379832 or XP_024380033	uncharacterized protein LOC112284343 [Physcomitrella patens] or alpha carbonic anhydrase 7-like [Physcomitrella patens]
**TRINITY_DN38568_c0_g1**	XP_024393797	uncharacterized protein LOC112291067 [Physcomitrella patens]
**TRINITY_DN50465_c0_g1**	XP_024363543	non-specific lipid-transfer protein 3-like [Physcomitrella patens]
**TRINITY_DN929_c0_g1**	XP_024395254	chalcone synthase 6-4-like [Physcomitrella patens] or ABU87504 2'-oxoalkylresorcinol synthase [Physcomitrella patens]
**TRINITY_DN32998_c0_g1**	XP_024363339	abscisic acid 8'-hydroxylase 3-like [Physcomitrella patens]
**TRINITY_DN32681_c2_g1**	PNR53501	hypothetical protein PHYPA_007176 [Physcomitrella patens]
**TRINITY_DN38263_c0_g2**	XP_024381396	uncharacterized protein LOC112285094 [Physcomitrella patens]
**TRINITY_DN11934_c0_g1**	XP_024368003	uncharacterized protein LOC112278633 [Physcomitrella patens]
**TRINITY_DN32215_c0_g1**	PNR26297	hypothetical protein PHYPA_030871 [Physcomitrella patens] / UDP-glycosyltransferase 83A1 (quercetin 3/7-O-glucosyltransferase activity)
**TRINITY_DN24061_c0_g1**	XP_024368984	GDSL esterase/lipase At4g16230-like [Physcomitrella patens]
**TRINITY_DN2561_c0_g1**	XP_024385936	probable pectinesterase 15 [Physcomitrella patens]
**TRINITY_DN55842_c0_g1**	XP_024377855	probable xyloglucan endotransglucosylase/hydrolase [Physcomitrella patens]
**TRINITY_DN61444_c0_g1**	XP_024388184	pathogen-related protein-like [Physcomitrella patens]

**TRINITY_DN6081_c0_g1**	XP_024395980	peroxidase 21-like isoform X1 [Physcomitrella patens] or XP_024395981 peroxidase 21-like isoform X2 [Physcomitrella patens]
**TRINITY_DN32215_c0_g2**	PNR26297	hypothetical protein PHYPA_030871 [Physcomitrella patens] / UDP-glycosyltransferase 83A1 (quercetin 3/7-O-glucosyltransferase activity)
**TRINITY_DN6494_c0_g1**	XP_024382012	GDSL esterase/lipase At5g03820-like [Physcomitrella patens]
**TRINITY_DN34803_c0_g1**	XP_024369838	uncharacterized protein LOC112279537 [Physcomitrella patens]<br>stigma-specific STIG1-like protein 1 [Helianthus annuus]
**TRINITY_DN38832_c0_g1**	PNR53501	hypothetical protein PHYPA_007176 [Physcomitrella patens]
**TRINITY_DN61594_c0_g1**	XP_024393924	germin-like protein 1-1 [Physcomitrella patens]
**TRINITY_DN26375_c0_g1**	XP_024382195	uncharacterized protein LOC112285518 [Physcomitrella patens]

For the mutant PTS_1+2 the top 25 list includes (yellow is part of the integration plasmid):

PTS_1+2 constitute the following contigs:

	325_1	Ole-LP4-PTS_1	Ole-PTS_1	Ole-PTS_2	PTS_1	PTS_2	LDAP-PTS_1	WT_1	WT_2
**TRINITY_DN44118_c21_g1**	171.545	497.621	2.988.097	2.589.358	2.583.480	2.661.563	5.737.899	0,263	0,424
**TRINITY_DN50091_c6_g1**	895.130	487.929	780.646	646.413	1.094.400	1.062.918	693.377	2.808	1.765
**TRINITY_DN34029_c1_g1**	112.605	637.773	2.189.608	1.961.225	537.918	541.147	1.549.930	1.323	0,504
**TRINITY_DN5532_c0_g1**	516.614	212.740	340.040	298.596	501.545	497.402	292.483	0,848	0,797
**TRINITY_DN49887_c0_g1**	407.396	409.522	364.535	345.914	429.405	420.770	226.078	0,455	0,494
**TRINITY_DN38568_c0_g1**	308.059	318.474	318.204	283.525	409.788	419.345	205.826	0,677	0,474
**TRINITY_DN17926_c0_g1**	354.116	412.054	361.551	317.894	310.472	319.082	20.319	0,313	0,091
**TRINITY_DN50465_c0_g1**	227.983	103.004	177.539	144.260	220.215	223.451	179.259	0,919	0,605
**TRINITY_DN32681_c2_g1**	108.301	77.126	93.735	76.088	143.615	141.120	67.175	0,424	0
**TRINITY_DN929_c0_g1**	143.373	129.993	111.031	107.181	139.801	135.391	75.454	0,222	0,081
**TRINITY_DN32998_c0_g1**	134.013	87.582	99.367	104.213	138.281	141.987	60.785	0,212	0,171
**TRINITY_DN11934_c0_g1**	128.166	44.605	80.074	74.416	126.998	123.885	63.216	0,313	0,504
**TRINITY_DN38263_c0_g2**	106.268	68.279	90.908	84.137	110.351	111.212	41.710	0,242	0,464
**TRINITY_DN32215_c0_g1**	95.022	69.967	79.254	63.245	98.750	96.966	62.665	0,121	0,121
**TRINITY_DN61444_c0_g1**	98.253	57.157	60.129	51.650	98.273	96.418	25.284	0,131	0
**TRINITY_DN24061_c0_g1**	96.773	63.175	75.713	67.884	89.969	90.281	54.965	0,131	0,161
**TRINITY_DN6081_c0_g1**	67.778	60.205	59.898	59.047	85.261	84.672	48.822	0,091	0,091
**TRINITY_DN32215_c0_g2**	68.518	51.676	51.502	46.742	75.428	77.280	45.688	0,121	0
**TRINITY_DN2000_c0_g1**	51.185	13.743	31.945	58.653	73.958	47.711	13.767	0	0
**TRINITY_DN55842_c0_g1**	64.985	102.100	62.336	60.075	71.991	74.699	72.283	0,162	0,04
**TRINITY_DN2561_c0_g1**	60.492	69.579	65.467	61.083	68.147	67.427	53.313	0,111	0
**TRINITY_DN6494_c0_g1**	66.006	42.212	43.946	44.408	59.715	59.805	29.984	0,03	0,05
**TRINITY_DN55646_c0_g1**	51.831	80.274	26.082	22.660	59.138	61.798	54.908	0,141	0,071
**TRINITY_DN38832_c0_g1**	53.207	70.423	40.195	40.767	57.659	54.037	30.307	0,061	0,252

**Contig in sequencing assembly**	P. patens NCBI #	**Description**
**TRINITY_DN44118_c21_g1**	ACK58633	kanomycin resistance protein [Cloning vector pHELLSGATE]
**TRINITY_DN50091_c6_g1**	PNR39672	hypothetical protein PHYPA_019951 [Physcomitrella patens]
**TRINITY_DN34029_c1_g1**	ALW83197	YFP-CcmM35 fusion protein (chloroplast) [synthetic construct]
**TRINITY_DN5532_c0_g1**	XP_024379832 or XP_024380033	uncharacterized protein LOC112284343 [Physcomitrella patens] or alpha carbonic anhydrase 7-like [Physcomitrella patens]
**TRINITY_DN49887_c0_g1**	XP_024393312	aldehyde oxidase GLOX-like [Physcomitrella patens]
**TRINITY_DN38568_c0_g1**	XP_024393797	uncharacterized protein LOC112291067 [Physcomitrella patens]
**TRINITY_DN17926_c0_g1**	XP_024365953 or XP_024368032	uncharacterized protein LOC112277638 isoform X2 [Physcomitrella patens] or alpha carbonic anhydrase 5-like [Physcomitrella patens]
**TRINITY_DN50465_c0_g1**	XP_024363543	non-specific lipid-transfer protein 3-like [Physcomitrella patens]
**TRINITY_DN32681_c2_g1**	PNR53501	hypothetical protein PHYPA_007176 [Physcomitrella patens]
**TRINITY_DN929_c0_g1**	XP_024395254	chalcone synthase 6-4-like [Physcomitrella patens] or ABU87504 2'-oxoalkylresorcinol synthase [Physcomitrella patens]
**TRINITY_DN32998_c0_g1**	XP_024363339	abscisic acid 8'-hydroxylase 3-like [Physcomitrella patens]
**TRINITY_DN11934_c0_g1**	XP_024368003	uncharacterized protein LOC112278633 [Physcomitrella patens]
**TRINITY_DN38263_c0_g2**	XP_024381396	uncharacterized protein LOC112285094 [Physcomitrella patens]
**TRINITY_DN32215_c0_g1**	PNR26297	hypothetical protein PHYPA_030871 [Physcomitrella patens] / UDP-glycosyltransferase 83A1 (quercetin 3/7-O-glucosyltransferase activity)
**TRINITY_DN61444_c0_g1**	XP_024388184	pathogen-related protein-like [Physcomitrella patens]
**TRINITY_DN24061_c0_g1**	XP_024368984	GDSL esterase/lipase At4g16230-like [Physcomitrella patens]
**TRINITY_DN6081_c0_g1**	XP_024395980	peroxidase 21-like isoform X1 [Physcomitrella patens] or XP_024395981 peroxidase 21-like isoform X2 [Physcomitrella patens]
**TRINITY_DN32215_c0_g2**	PNR26297	hypothetical protein PHYPA_030871 [Physcomitrella patens] / UDP-glycosyltransferase 83A1 (quercetin 3/7-O-glucosyltransferase activity)

**TRINITY_DN2000_c0_g1**	PNR47760	hypothetical protein PHYPA_012233 [Physcomitrella patens]
**TRINITY_DN55842_c0_g1**	XP_024377855	probable xyloglucan endotransglucosylase/hydrolase [Physcomitrella patens]
**TRINITY_DN2561_c0_g1**	XP_024385936	probable pectinesterase 15 [Physcomitrella patens]
**TRINITY_DN6494_c0_g1**	XP_024382012	GDSL esterase/lipase At5g03820-like [Physcomitrella patens]
**TRINITY_DN55646_c0_g1**	XP_024377855	probable xyloglucan endotransglucosylase/hydrolase [Physcomitrella patens]
**TRINITY_DN38832_c0_g1**	PNR53501	hypothetical protein PHYPA_007176 [Physcomitrella patens]

For the mutant AtLDAP_PTS_1 the top 25 list includes (yellow is part of the integration plasmid):

LDAP_PTS_1 constitute the following contigs:

	325_1	Ole-LP4-PTS_1	Ole-PTS_1	Ole-PTS_2	PTS_1	PTS_2	LDAP-PTS_1	WT_1	WT_2
**TRINITY_DN44118_c21_g1**	171.545	497.621	2.988.097	2.589.358	2.583.480	2.661.563	5.737.899	0,263	0,424
**TRINITY_DN34029_c1_g1**	112.605	637.773	2.189.608	1.961.225	537.918	541.147	1.549.930	1.323	0,504
**TRINITY_DN50091_c6_g1**	895.130	487.929	780.646	646.413	1.094.400	1.062.918	693.377	2.808	1.765
**TRINITY_DN5532_c0_g1**	516.614	212.740	340.040	298.596	501.545	497.402	292.483	0,848	0,797
**TRINITY_DN34534_c0_g1**	0	0,02	2.942	2.834	0,834	0,687	270.067	0	0,04
**TRINITY_DN49887_c0_g1**	407.396	409.522	364.535	345.914	429.405	420.770	226.078	0,455	0,494
**TRINITY_DN38568_c0_g1**	308.059	318.474	318.204	283.525	409.788	419.345	205.826	0,677	0,474
**TRINITY_DN50465_c0_g1**	227.983	103.004	177.539	144.260	220.215	223.451	179.259	0,919	0,605
**TRINITY_DN929_c0_g1**	143.373	129.993	111.031	107.181	139.801	135.391	75.454	0,222	0,081
**TRINITY_DN55842_c0_g1**	64.985	102.100	62.336	60.075	71.991	74.699	72.283	0,162	0,04
**TRINITY_DN32681_c2_g1**	108.301	77.126	93.735	76.088	143.615	141.120	67.175	0,424	0
**TRINITY_DN11934_c0_g1**	128.166	44.605	80.074	74.416	126.998	123.885	63.216	0,313	0,504
**TRINITY_DN32215_c0_g1**	95.022	69.967	79.254	63.245	98.750	96.966	62.665	0,121	0,121
**TRINITY_DN32998_c0_g1**	134.013	87.582	99.367	104.213	138.281	141.987	60.785	0,212	0,171
**TRINITY_DN24061_c0_g1**	96.773	63.175	75.713	67.884	89.969	90.281	54.965	0,131	0,161
**TRINITY_DN55646_c0_g1**	51.831	80.274	26.082	22.660	59.138	61.798	54.908	0,141	0,071
**TRINITY_DN2561_c0_g1**	60.492	69.579	65.467	61.083	68.147	67.427	53.313	0,111	0
**TRINITY_DN6081_c0_g1**	67.778	60.205	59.898	59.047	85.261	84.672	48.822	0,091	0,091
**TRINITY_DN32215_c0_g2**	68.518	51.676	51.502	46.742	75.428	77.280	45.688	0,121	0
**TRINITY_DN38263_c0_g2**	106.268	68.279	90.908	84.137	110.351	111.212	41.710	0,242	0,464
**TRINITY_DN16400_c0_g1**	46.974	53.374	29.781	30.364	42.293	44.005	39.175	0,111	0,111
**TRINITY_DN26375_c0_g1**	36.083	27.158	33.911	31.843	40.783	42.739	39.004	0	0
**TRINITY_DN34803_c0_g1**	53.395	18.301	43.673	31.661	51.997	51.616	35.747	0,051	0,212
**TRINITY_DN38832_c0_g1**	53.207	70.423	40.195	40.767	57.659	54.037	30.307	0,061	0,252
**TRINITY_DN6494_c0_g1**	66.006	42.212	43.946	44.408	59.715	59.805	29.984	0,03	0,05

Contig in sequencing assembly	P. patens NCBI #	Description
**TRINITY_DN44118_c21_g1**	ACK58633	kanomycin resistance protein [Cloning vector pHELLSGATE]
**TRINITY_DN34029_c1_g1**	ALW83197	YFP-CcmM35 fusion protein (chloroplast) [synthetic construct]
**TRINITY_DN50091_c6_g1**	PNR39672	hypothetical protein PHYPA_019951 [Physcomitrella patens]
**TRINITY_DN5532_c0_g1**	XP_024379832 or XP_024380033	uncharacterized protein LOC112284343 [Physcomitrella patens] or alpha carbonic anhydrase 7-like [Physcomitrella patens]
**TRINITY_DN34534_c0_g1**	AQT31662	neomycin phosphotransferase II [Binary vector pBI121]
**TRINITY_DN49887_c0_g1**	XP_024393312	aldehyde oxidase GLOX-like [Physcomitrella patens]
**TRINITY_DN38568_c0_g1**	XP_024393797	uncharacterized protein LOC112291067 [Physcomitrella patens]
**TRINITY_DN50465_c0_g1**	XP_024363543	non-specific lipid-transfer protein 3-like [Physcomitrella patens]
**TRINITY_DN929_c0_g1**	XP_024395254	chalcone synthase 6-4-like [Physcomitrella patens] or ABU87504 2'-oxoalkylresorcinol synthase [Physcomitrella patens]
**TRINITY_DN55842_c0_g1**	XP_024377855	probable xyloglucan endotransglucosylase/hydrolase [Physcomitrella patens]
**TRINITY_DN32681_c2_g1**	PNR53501	hypothetical protein PHYPA_007176 [Physcomitrella patens]
**TRINITY_DN11934_c0_g1**	XP_024368003	uncharacterized protein LOC112278633 [Physcomitrella patens]
**TRINITY_DN32215_c0_g1**	PNR26297	hypothetical protein PHYPA_030871 [Physcomitrella patens] / UDP-glycosyltransferase 83A1 (quercetin 3/7-O-glucosyltransferase activity)
**TRINITY_DN32998_c0_g1**	XP_024363339	abscisic acid 8'-hydroxylase 3-like [Physcomitrella patens]
**TRINITY_DN24061_c0_g1**	XP_024368984	GDSL esterase/lipase At4g16230-like [Physcomitrella patens]
**TRINITY_DN55646_c0_g1**	XP_024375379	non-specific lipid-transfer protein 2G-like [Physcomitrella patens]
**TRINITY_DN2561_c0_g1**	XP_024385936	probable pectinesterase 15 [Physcomitrella patens]
**TRINITY_DN6081_c0_g1**	XP_024395980	peroxidase 21-like isoform X1 [Physcomitrella patens] or XP_024395981 peroxidase 21-like isoform X2 [Physcomitrella patens]

**TRINITY_DN32215_c0_g2**	PNR26297	hypothetical protein PHYPA_030871 [Physcomitrella patens] / UDP-glycosyltransferase 83A1 (quercetin 3/7-O-glucosyltransferase activity)
**TRINITY_DN38263_c0_g2**	XP_024381396	uncharacterized protein LOC112285094 [Physcomitrella patens]
**TRINITY_DN16400_c0_g1**	XP_024395390	receptor-like protein 51 [Physcomitrella patens]
**TRINITY_DN26375_c0_g1**	XP_024382195	uncharacterized protein LOC112285518 [Physcomitrella patens]
**TRINITY_DN34803_c0_g1**	XP_024369838	uncharacterized protein LOC112279537 [Physcomitrella patens]<br>stigma-specific STIG1-like protein 1 [Helianthus annuus]
**TRINITY_DN38832_c0_g1**	PNR53501	hypothetical protein PHYPA_007176 [Physcomitrella patens]
**TRINITY_DN6494_c0_g1**	XP_024382012	GDSL esterase/lipase At5g03820-like [Physcomitrella patens]

25 most down regulated genes in the mutants in comparison with WT.

	325_1	Ole-LP4-PTS_1	Ole-PTS_1	Ole-PTS_2	PTS_1	PTS_2	LDAP-PTS_1	WT_1	WT_2
**TRINITY_DN6295_c0_g1**	0,761	0,318	0,347	0,759	0,775	0,638	5.583	442.419	442.551
**TRINITY_DN26229_c0_g1**	0	0	0	0,077	0,278	0	10.444	294.842	295.363
**TRINITY_DN50057_c0_g1**	0,25	0,02	0,284	0,134	0,079	0	2.488	257.611	250.164
**TRINITY_DN12927_c0_g1**	0,063	0	0	0	0	0	0,902	153.759	145.674
**TRINITY_DN27466_c1_g1**	2.053	0,258	0,83	0,865	0,954	1.494	8.346	117.306	120.780
**TRINITY_DN38313_c0_g1**	0,208	1.668	0,305	0,048	0,348	0,299	3.741	110.196	111.984
**TRINITY_DN38368_c0_g1**	0	0	0	0	0	0	1.889	99.469	94.958
**TRINITY_DN45734_c0_g1**	0,646	0,745	0,368	0,528	0,288	0,279	4.121	92.045	82.652
**TRINITY_DN39595_c0_g1**	0,49	0,139	0,399	0,413	0,656	0,697	0,826	70.814	69.287
**TRINITY_DN30687_c0_g1**	0	0	1.639	0	3.049	1.315	8.745	45.371	45.956
**TRINITY_DN34516_c3_g1**	0,354	7.855	9.521	3.593	17.452	0	9.599	43.593	29.010
**TRINITY_DN29028_c0_g2**	0	2.800	4.540	3.218	2.811	1.215	2.687	38.069	47.297
**TRINITY_DN20134_c2_g1**	0,104	0,099	0	0,058	0,05	0,08	0,911	37.442	37.806
**TRINITY_DN5993_c0_g1**	0	0	0,053	0,058	0,159	0,08	0,399	30.746	32.631
**TRINITY_DN20064_c0_g1**	0,25	0	0,578	0,202	1.152	0,936	0,826	27.796	29.252
**TRINITY_DN23122_c0_g1**	0	7.368	4.392	4.678	4.142	3.477	10.947	27.110	37.059
**TRINITY_DN20262_c0_g1**	0,042	0,129	0,021	0,048	0,089	0,07	0,475	26.988	26.902
**TRINITY_DN18350_c0_g1**	0,042	0,159	0,389	0,202	0,04	0,239	1.519	25.493	26.095
**TRINITY_DN24155_c0_g1**	0	0	0,284	0	0,209	0,219	1.035	23.655	24.108
**TRINITY_DN12351_c0_g1**	0,073	0,467	0,221	0	0,089	0,11	0,266	22.090	25.590
**TRINITY_DN33997_c0_g3**	0	0,566	0,914	0,634	0,298	0,707	2.450	21.443	18.862
**TRINITY_DN23815_c0_g1**	0,49	0,199	0,147	0	0,785	0,936	1.424	21.100	21.293
**TRINITY_DN32661_c0_g1**	0,855	0,238	0,504	0,384	0	0	5.564	19.837	17.279
**TRINITY_DN46340_c0_g1**	0,177	0	0,273	0,192	0,179	0,289	0,589	19.756	20.365

**Contig in sequencing assembly**	P. patens NCBI #	**Description**
**TRINITY_DN6295_c0_g1**	XP_024375887	chlorophyll a-b binding protein of LHCII type 1-like [Physcomitrella patens]
**TRINITY_DN26229_c0_g1**	XP_024400099	copper chaperone for superoxide dismutase, chloroplastic/cytosolic-like isoform X2 [Physcomitrella patens]
**TRINITY_DN50057_c0_g1**	XP_024367800	ferric reduction oxidase 6-like [Physcomitrella patens]
**TRINITY_DN12927_c0_g1**	XP_024362229	germin-like protein 9-3 [Physcomitrella patens]
**TRINITY_DN27466_c1_g1**	XP_024399688	phospho-2-dehydro-3-deoxyheptonate aldolase 2, chloroplastic-like isoform X3 [Physcomitrella patens]
**TRINITY_DN38313_c0_g1**	XP_024360503	GDSL esterase/lipase At4g01130-like [Physcomitrella patens]
**TRINITY_DN38368_c0_g1**	PNR36232	hypothetical protein PHYPA_022083 [Physcomitrella patens]
**TRINITY_DN45734_c0_g1**	PNR34789	hypothetical protein PHYPA_022687 [Physcomitrella patens] (chaperone dnaJ8, chloroplastic)
**TRINITY_DN39595_c0_g1**	XP_024358907	formin-1-like [Physcomitrella patens]
**TRINITY_DN30687_c0_g1**		Unknown, no hits found in BLAST
**TRINITY_DN34516_c3_g1**	XP_024359073	chlorophyll a-b binding protein, chloroplastic-like [Physcomitrella patens]
**TRINITY_DN29028_c0_g2**		Unknown, no hits found in BLAST
**TRINITY_DN20134_c2_g1**	XP_024403288	polyphenol oxidase, chloroplastic-like [Physcomitrella patens]
**TRINITY_DN5993_c0_g1**		Unknown, no hits found in BLAST
**TRINITY_DN20064_c0_g1**	XP_024392911	protein LURP-one-related 15-like [Physcomitrella patens]
**TRINITY_DN23122_c0_g1**	XP_024378832	dirigent protein 19-like [Physcomitrella patens]
**TRINITY_DN20262_c0_g1**	PNR36153	hypothetical protein PHYPA_022004 [Physcomitrella patens]
**TRINITY_DN18350_c0_g1**	XP_024397347	enolase 1, chloroplastic-like [Physcomitrella patens]
**TRINITY_DN24155_c0_g1**	PNR55678	hypothetical protein PHYPA_006575 [Physcomitrella patens]
**TRINITY_DN12351_c0_g1**	XP_024367311	copper transporter 5.1-like [Physcomitrella patens]
**TRINITY_DN33997_c0_g3**		Unknown, no hits found in BLAST

**TRINITY_DN23815_c0_g1**	XP_024360335	1-Cys peroxiredoxin-like [Physcomitrella patens]
**TRINITY_DN32661_c0_g1**	PNR52172, PNR35977	hypothetical protein [Physcomitrella patens]
**TRINITY_DN46340_c0_g1**	PNR36032	hypothetical protein PHYPA_021882 [Physcomitrella patens]
