# Supplementary figures and images for: Connecting moss lipid droplets to patchoulol biosynthesis
Source: PLoS One. 2020 Dec 7;15(12):e0243620. doi: 10.1371/journal.pone.0243620 (PMC7721168; doi:10.1371/journal.pone.0243620)

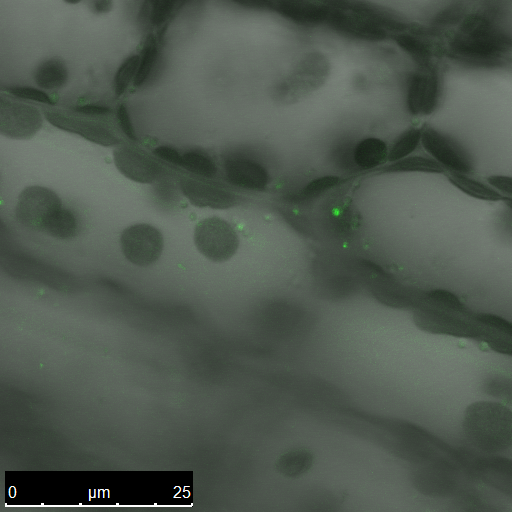

Supplement: S3 File — (ZIP) [file pone.0243620.s003.zip › 325 OE/Experiment_325 OE begin 99.tif]

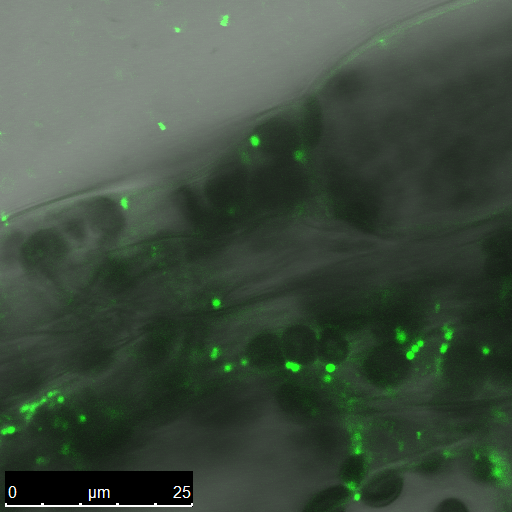

Supplement: S3 File — (ZIP) [file pone.0243620.s003.zip › 325 OE/Experiment_325 OE End 133.tif]

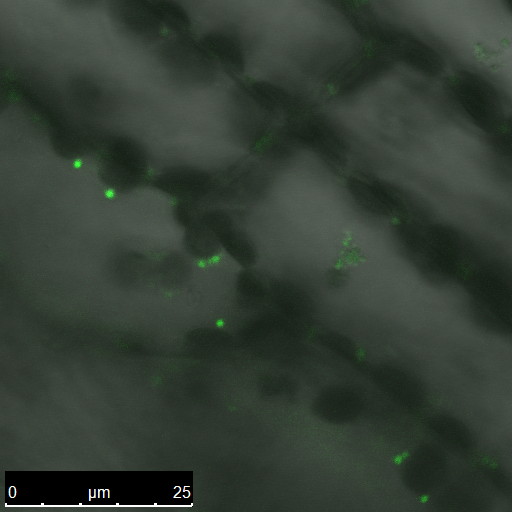

Supplement: S3 File — (ZIP) [file pone.0243620.s003.zip › 325 OE/Experiment_Image101.tif]

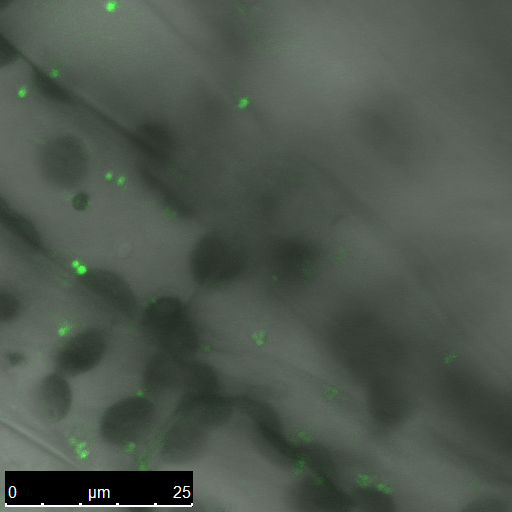

Supplement: S3 File — (ZIP) [file pone.0243620.s003.zip › 325 OE/Experiment_Image103.tif]

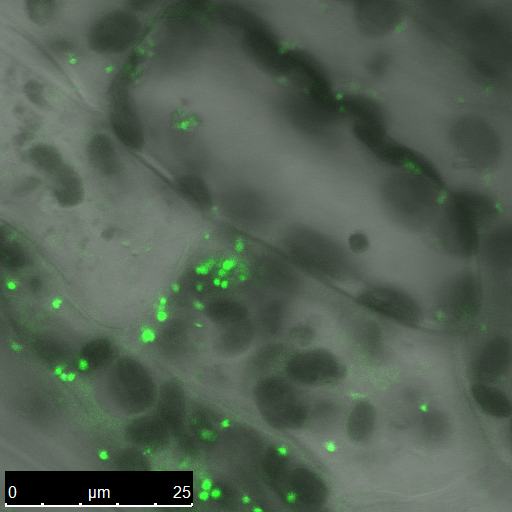

Supplement: S3 File — (ZIP) [file pone.0243620.s003.zip › 325 OE/Experiment_Image105.tif]

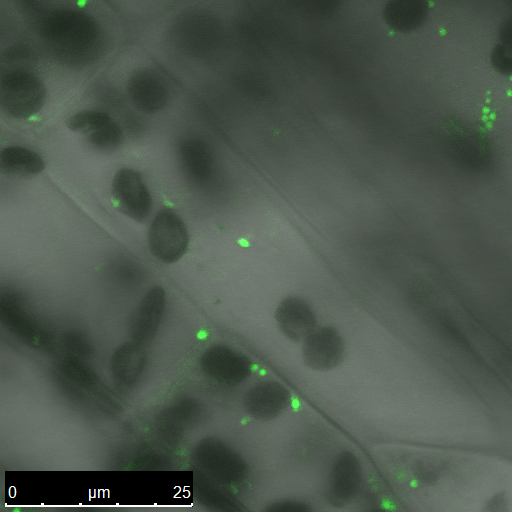

Supplement: S3 File — (ZIP) [file pone.0243620.s003.zip › 325 OE/Experiment_Image107.tif]

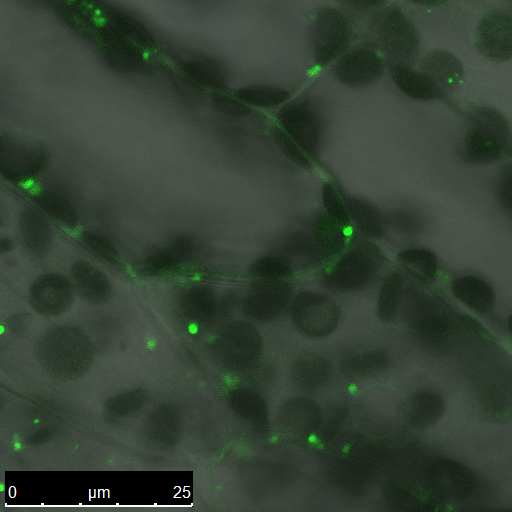

Supplement: S3 File — (ZIP) [file pone.0243620.s003.zip › 325 OE/Experiment_Image109.tif]

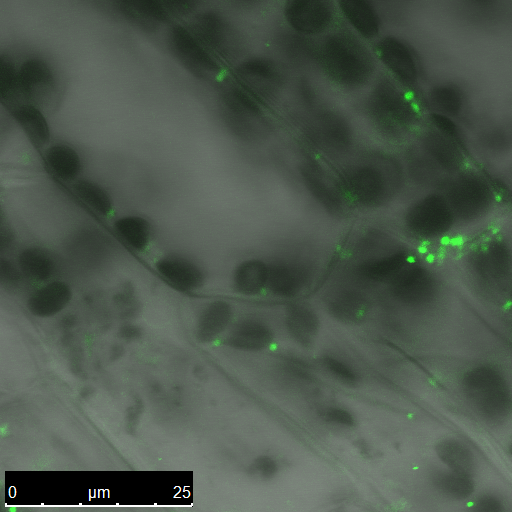

Supplement: S3 File — (ZIP) [file pone.0243620.s003.zip › 325 OE/Experiment_Image111.tif]

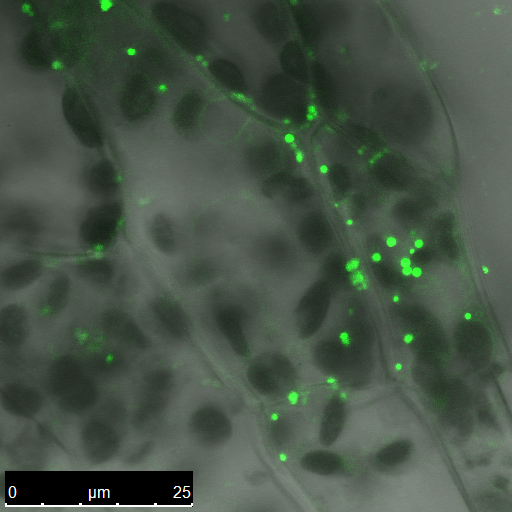

Supplement: S3 File — (ZIP) [file pone.0243620.s003.zip › 325 OE/Experiment_Image113.tif]

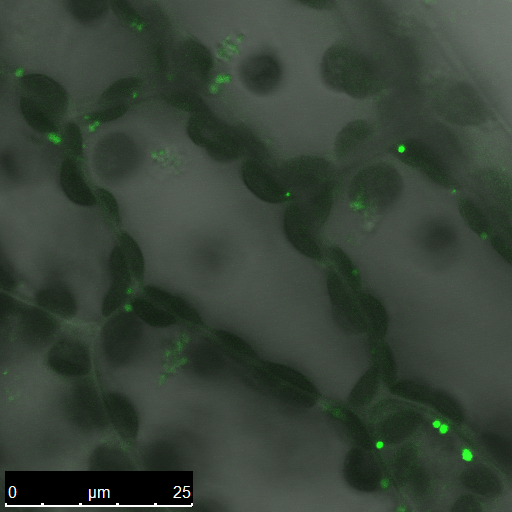

Supplement: S3 File — (ZIP) [file pone.0243620.s003.zip › 325 OE/Experiment_Image115.tif]

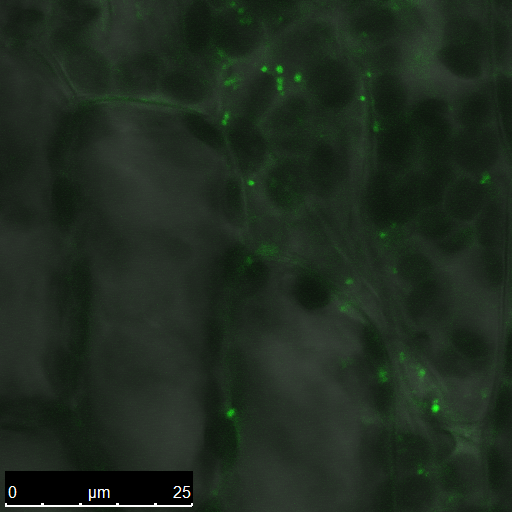

Supplement: S3 File — (ZIP) [file pone.0243620.s003.zip › 325 OE/Experiment_Image117.tif]

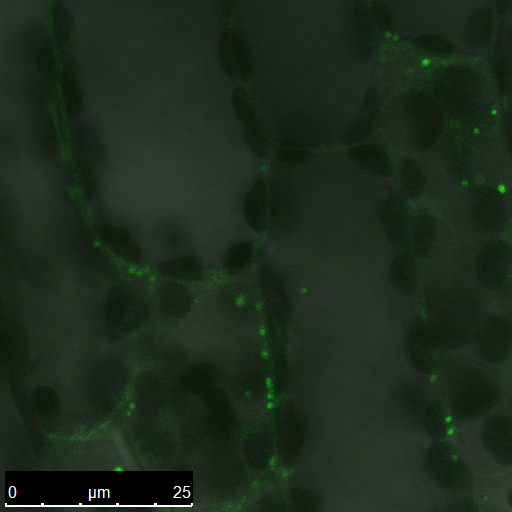

Supplement: S3 File — (ZIP) [file pone.0243620.s003.zip › 325 OE/Experiment_Image119.tif]

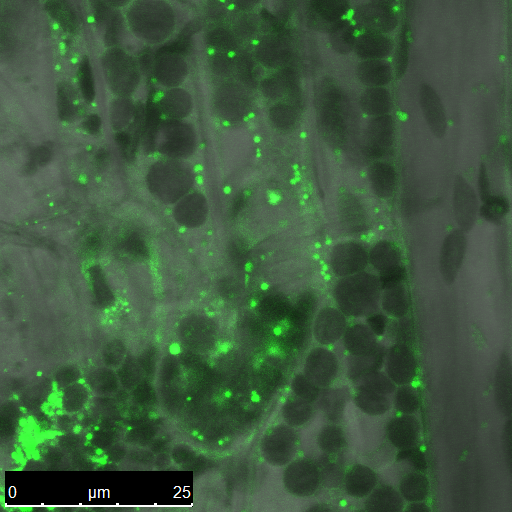

Supplement: S3 File — (ZIP) [file pone.0243620.s003.zip › 325 OE/Experiment_Image121.tif]

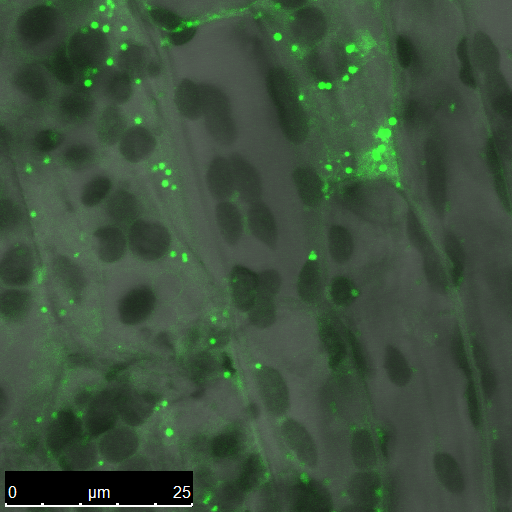

Supplement: S3 File — (ZIP) [file pone.0243620.s003.zip › 325 OE/Experiment_Image123.tif]

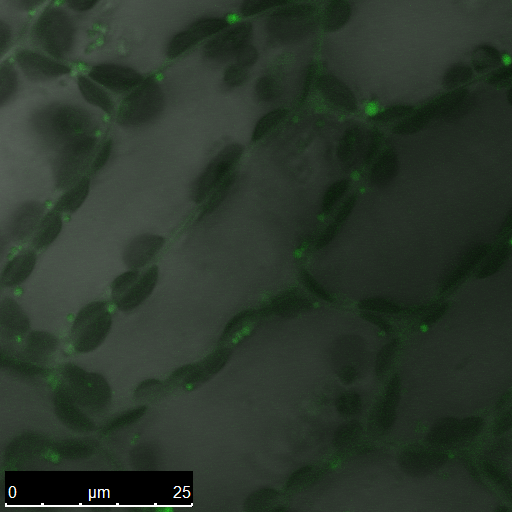

Supplement: S3 File — (ZIP) [file pone.0243620.s003.zip › 325 OE/Experiment_Image127.tif]

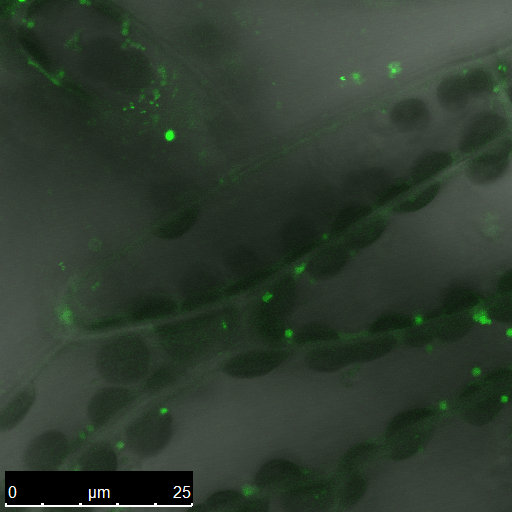

Supplement: S3 File — (ZIP) [file pone.0243620.s003.zip › 325 OE/Experiment_Image129.tif]

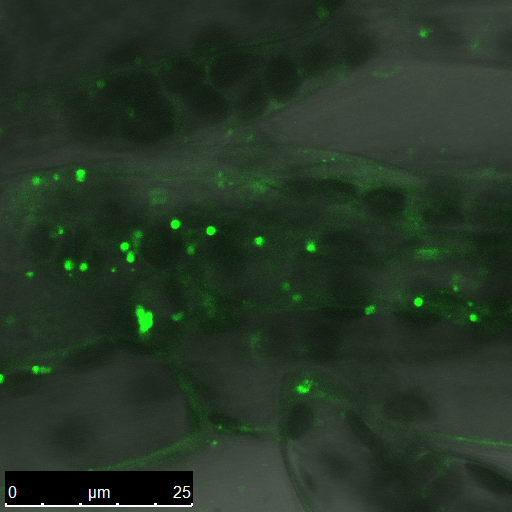

Supplement: S3 File — (ZIP) [file pone.0243620.s003.zip › 325 OE/Experiment_Image131.tif]

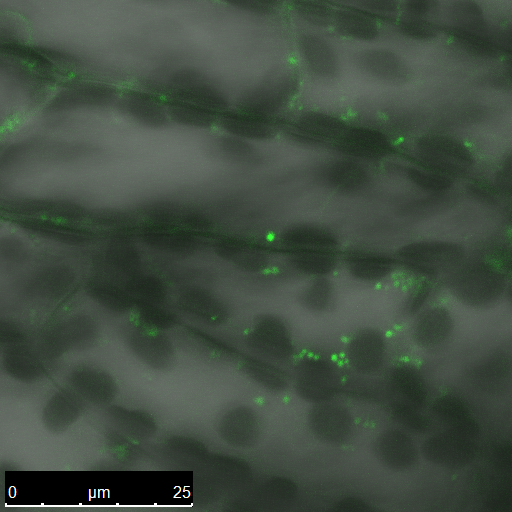

Supplement: S3 File — (ZIP) [file pone.0243620.s003.zip › 325-PTS/Experiment_325 PTS last 97.tif]

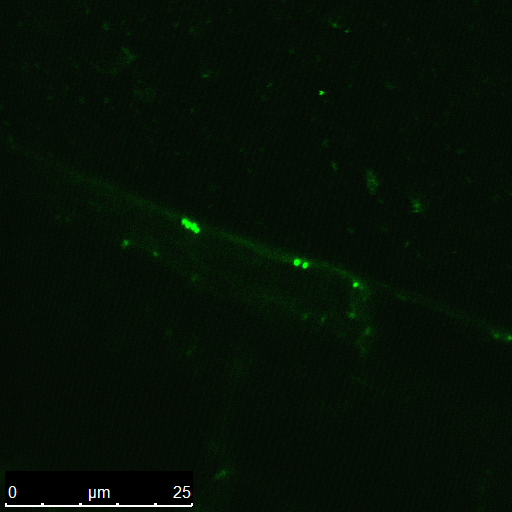

Supplement: S3 File — (ZIP) [file pone.0243620.s003.zip › 325-PTS/Experiment_Image054 325 PTS begining.tif]

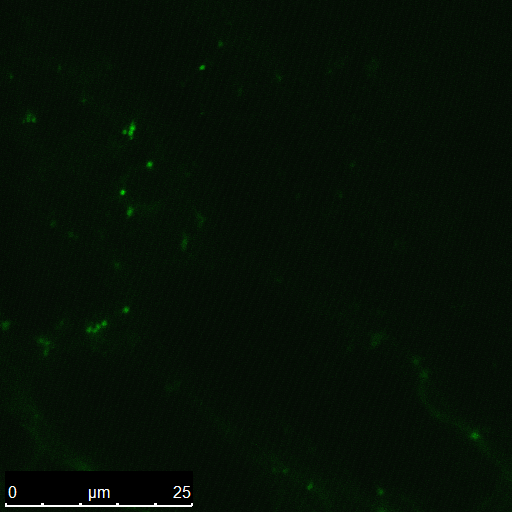

Supplement: S3 File — (ZIP) [file pone.0243620.s003.zip › 325-PTS/Experiment_Image056.tif]

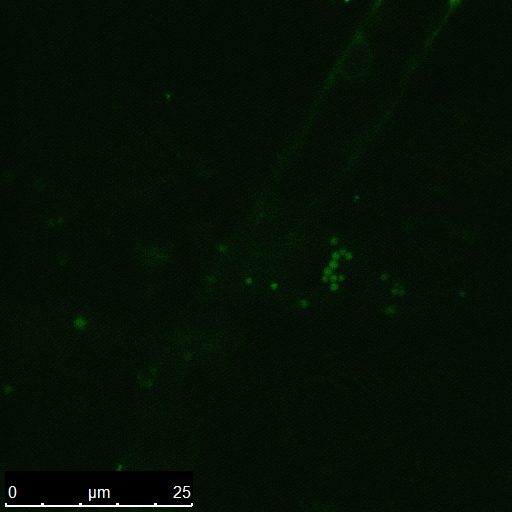

Supplement: S3 File — (ZIP) [file pone.0243620.s003.zip › 325-PTS/Experiment_Image059.tif]

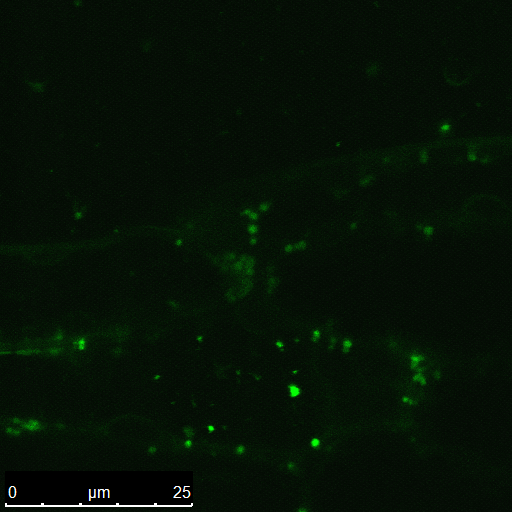

Supplement: S3 File — (ZIP) [file pone.0243620.s003.zip › 325-PTS/Experiment_Image061.tif]

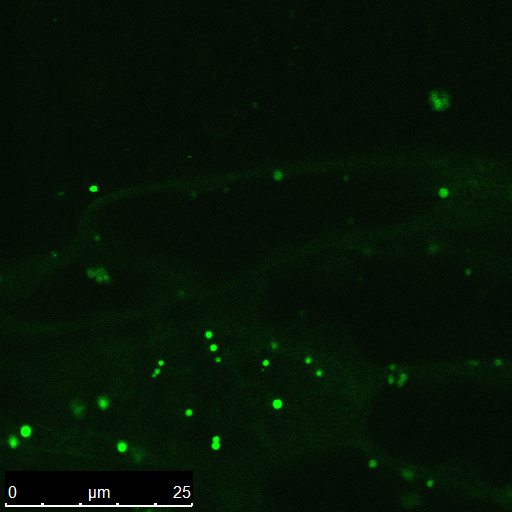

Supplement: S3 File — (ZIP) [file pone.0243620.s003.zip › 325-PTS/Experiment_Image065.tif]

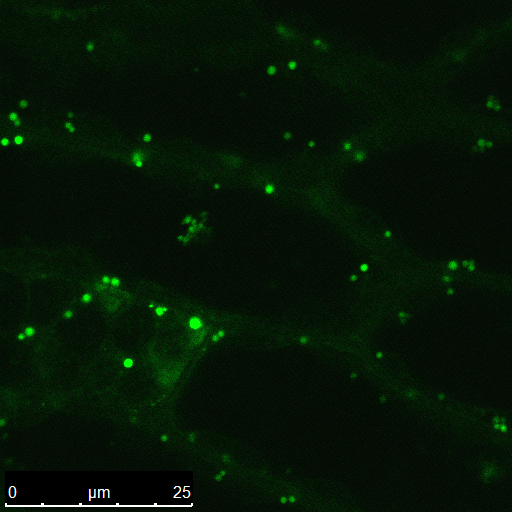

Supplement: S3 File — (ZIP) [file pone.0243620.s003.zip › 325-PTS/Experiment_Image067.tif]

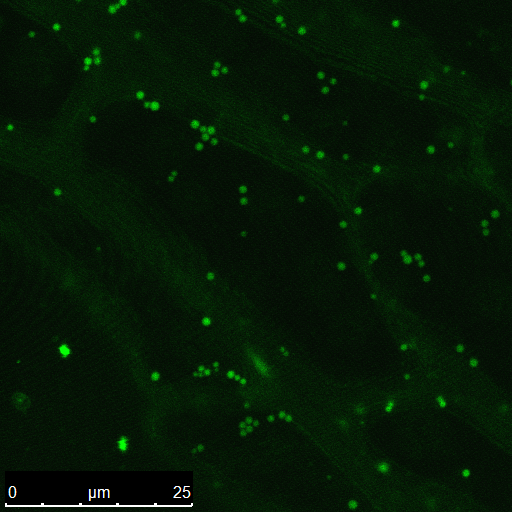

Supplement: S3 File — (ZIP) [file pone.0243620.s003.zip › 325-PTS/Experiment_Image069.tif]

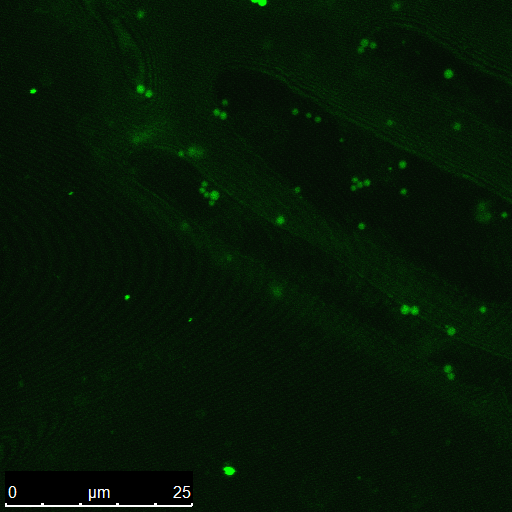

Supplement: S3 File — (ZIP) [file pone.0243620.s003.zip › 325-PTS/Experiment_Image071.tif]

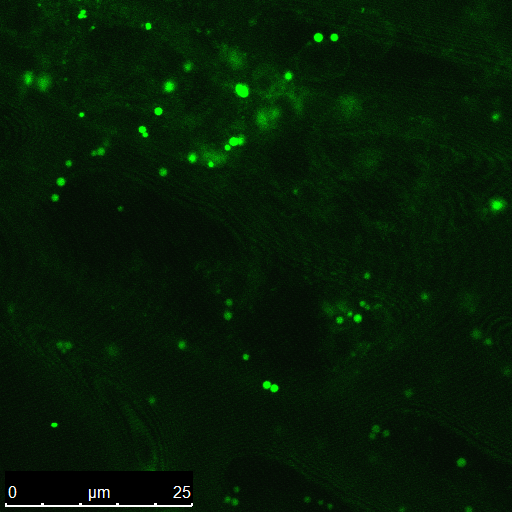

Supplement: S3 File — (ZIP) [file pone.0243620.s003.zip › 325-PTS/Experiment_Image073.tif]

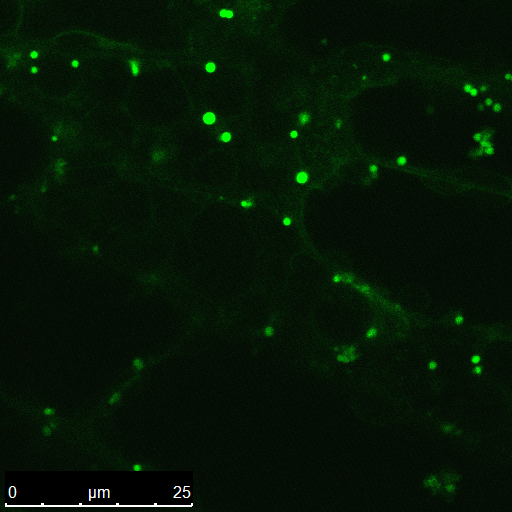

Supplement: S3 File — (ZIP) [file pone.0243620.s003.zip › 325-PTS/Experiment_Image075.tif]

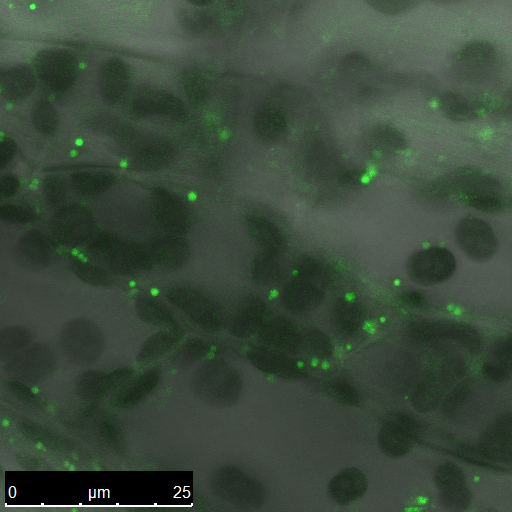

Supplement: S3 File — (ZIP) [file pone.0243620.s003.zip › 325-PTS/Experiment_Image077.tif]

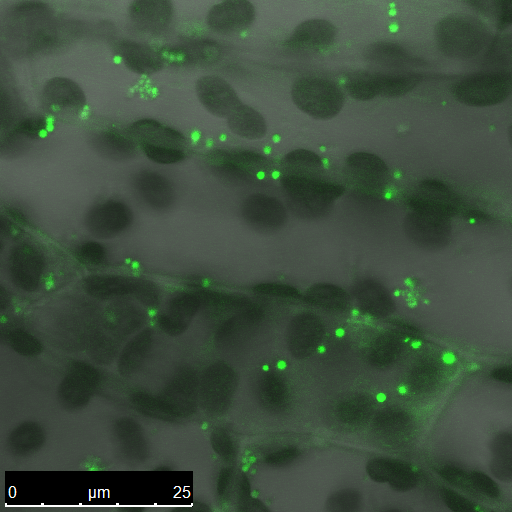

Supplement: S3 File — (ZIP) [file pone.0243620.s003.zip › 325-PTS/Experiment_Image079.tif]

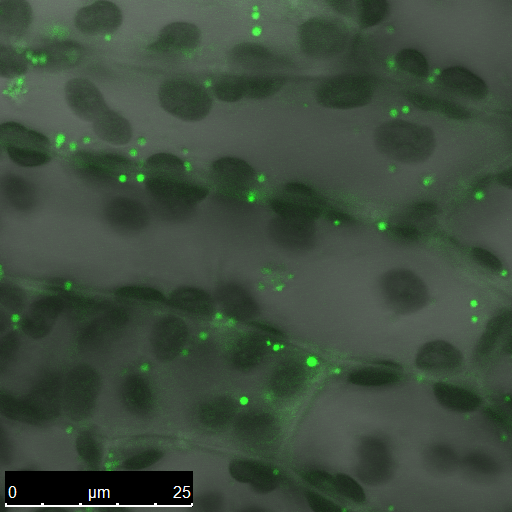

Supplement: S3 File — (ZIP) [file pone.0243620.s003.zip › 325-PTS/Experiment_Image081.tif]

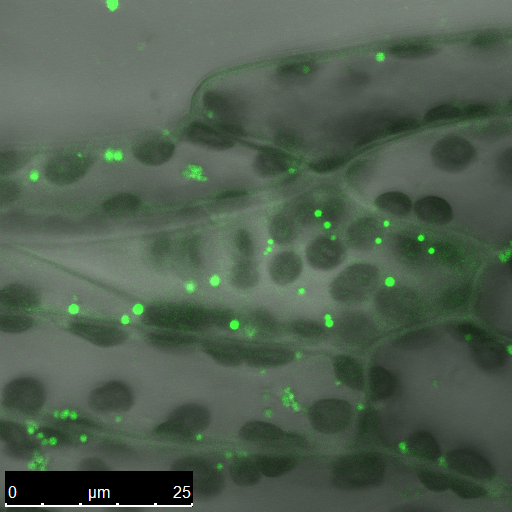

Supplement: S3 File — (ZIP) [file pone.0243620.s003.zip › 325-PTS/Experiment_Image083.tif]

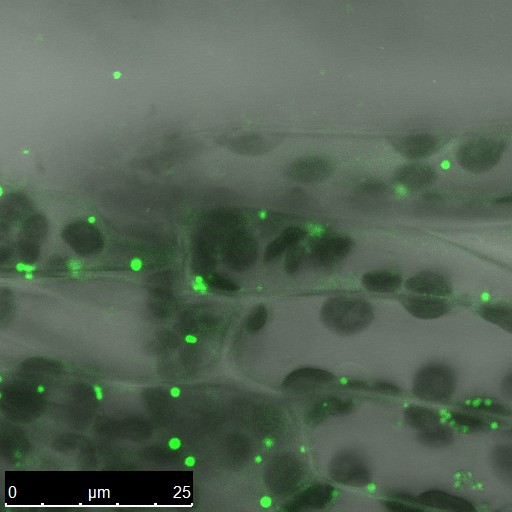

Supplement: S3 File — (ZIP) [file pone.0243620.s003.zip › 325-PTS/Experiment_Image085.tif]

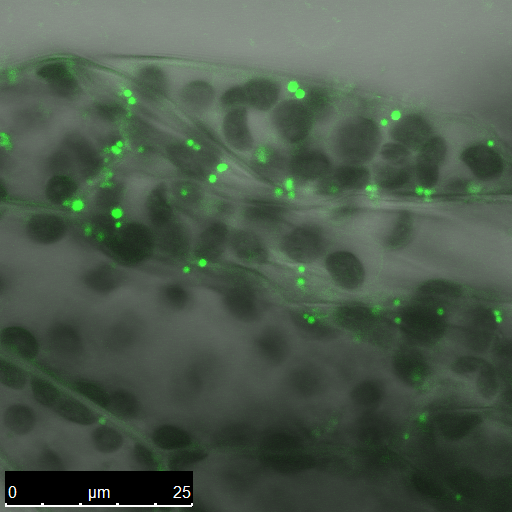

Supplement: S3 File — (ZIP) [file pone.0243620.s003.zip › 325-PTS/Experiment_Image087.tif]

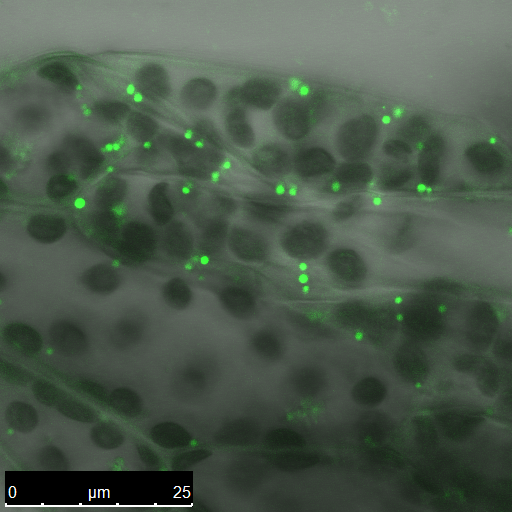

Supplement: S3 File — (ZIP) [file pone.0243620.s003.zip › 325-PTS/Experiment_Image089.tif]

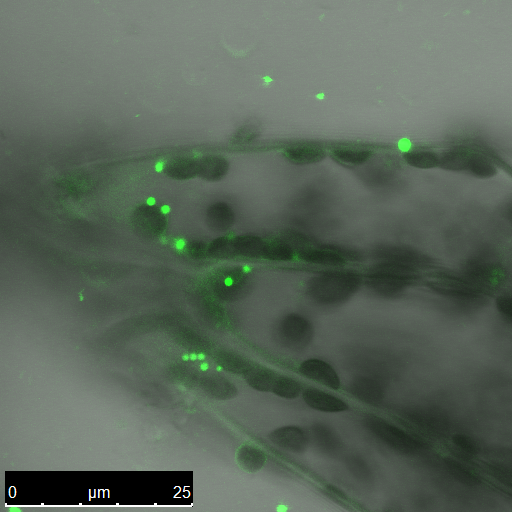

Supplement: S3 File — (ZIP) [file pone.0243620.s003.zip › 325-PTS/Experiment_Image091.tif]

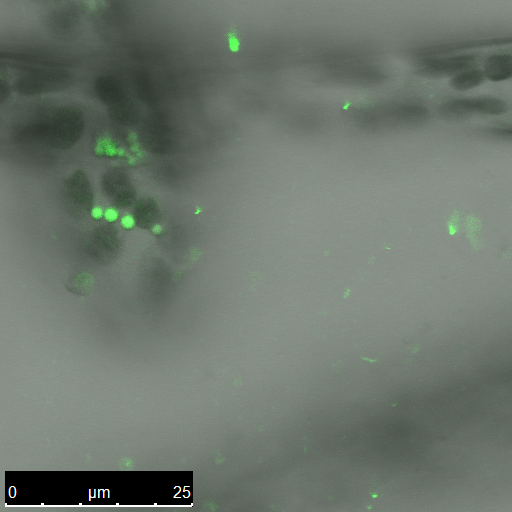

Supplement: S3 File — (ZIP) [file pone.0243620.s003.zip › 325-PTS/Experiment_Image093.tif]

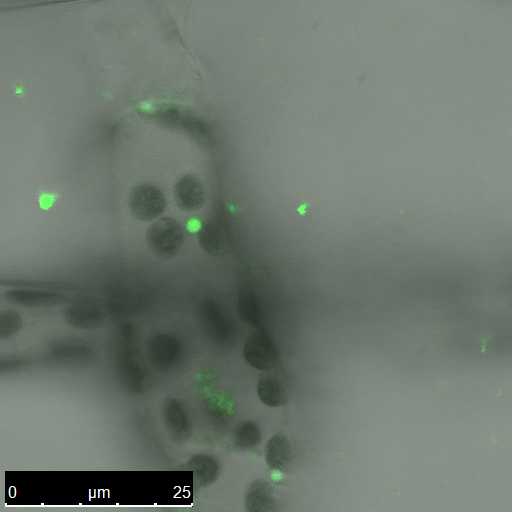

Supplement: S3 File — (ZIP) [file pone.0243620.s003.zip › 325-PTS/Experiment_Image095.tif]

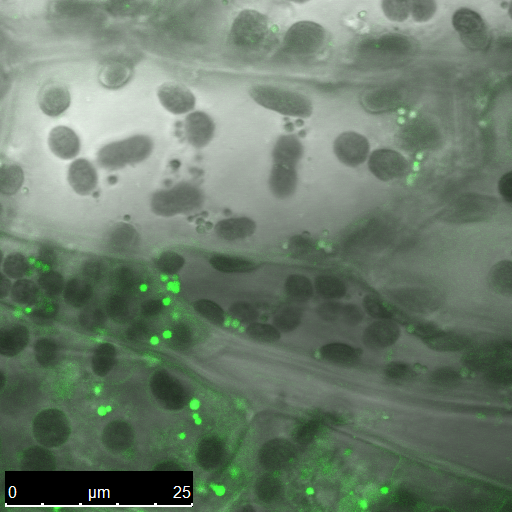

Supplement: S3 File — (ZIP) [file pone.0243620.s003.zip › 325-Venus/Experiment_Image183 325V begin.tif]

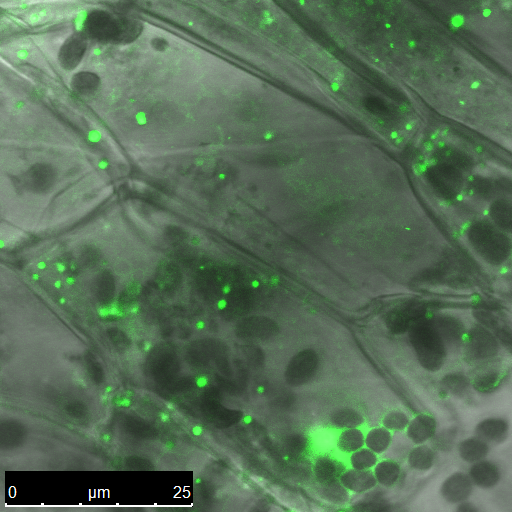

Supplement: S3 File — (ZIP) [file pone.0243620.s003.zip › 325-Venus/Experiment_Image185.tif]

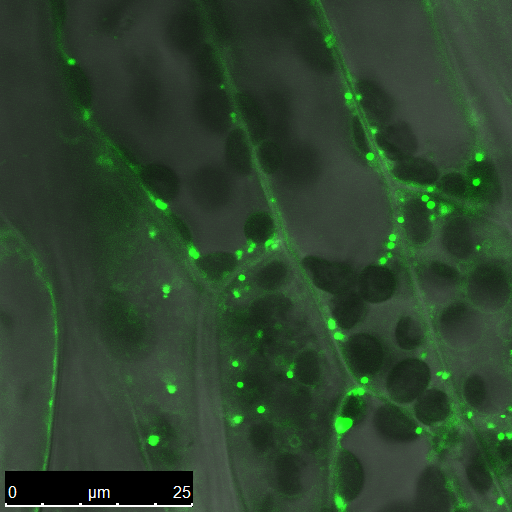

Supplement: S3 File — (ZIP) [file pone.0243620.s003.zip › 325-Venus/Experiment_Image187.tif]

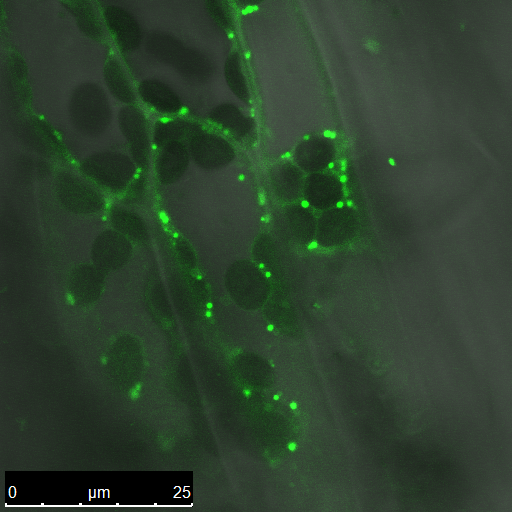

Supplement: S3 File — (ZIP) [file pone.0243620.s003.zip › 325-Venus/Experiment_Image189.tif]

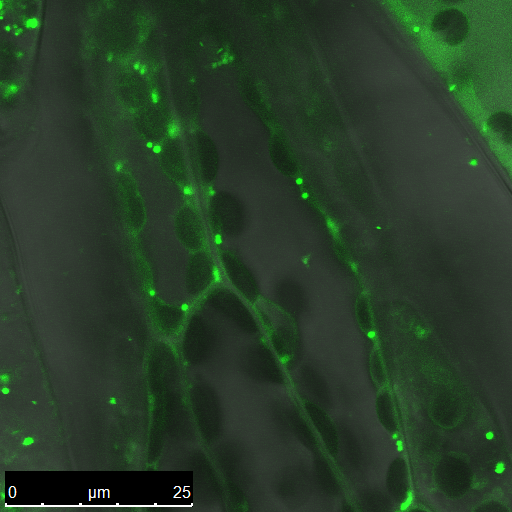

Supplement: S3 File — (ZIP) [file pone.0243620.s003.zip › 325-Venus/Experiment_Image191.tif]

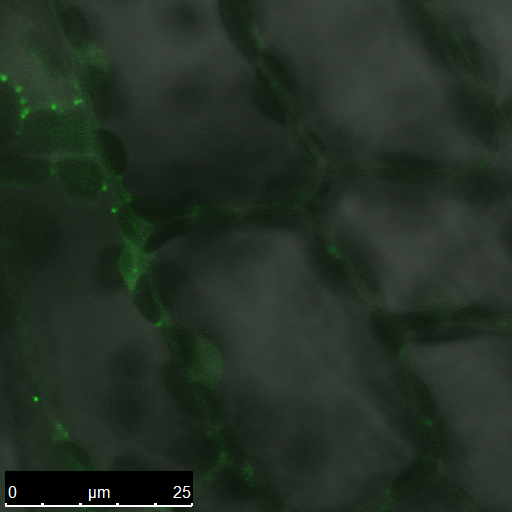

Supplement: S3 File — (ZIP) [file pone.0243620.s003.zip › 325-Venus/Experiment_Image193.tif]

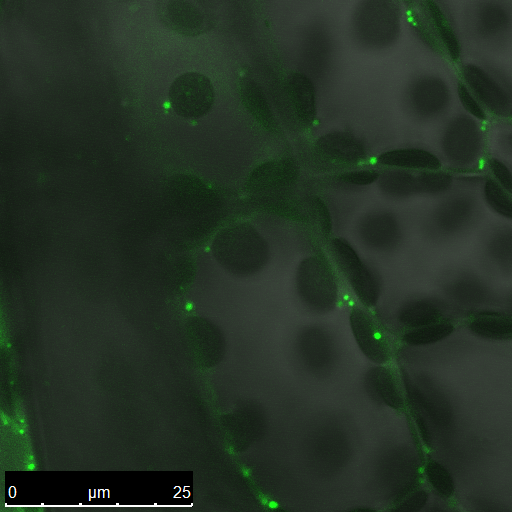

Supplement: S3 File — (ZIP) [file pone.0243620.s003.zip › 325-Venus/Experiment_Image195.tif]

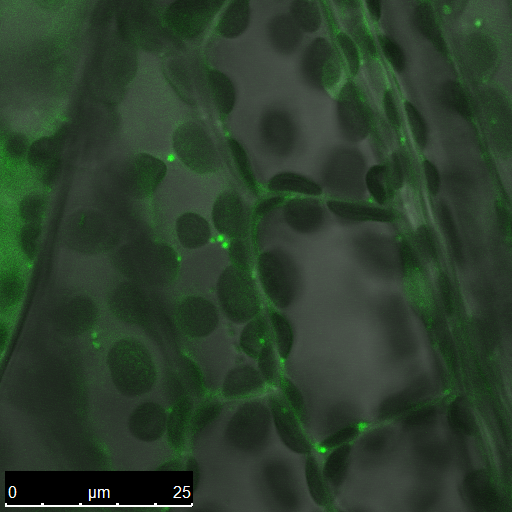

Supplement: S3 File — (ZIP) [file pone.0243620.s003.zip › 325-Venus/Experiment_Image197.tif]

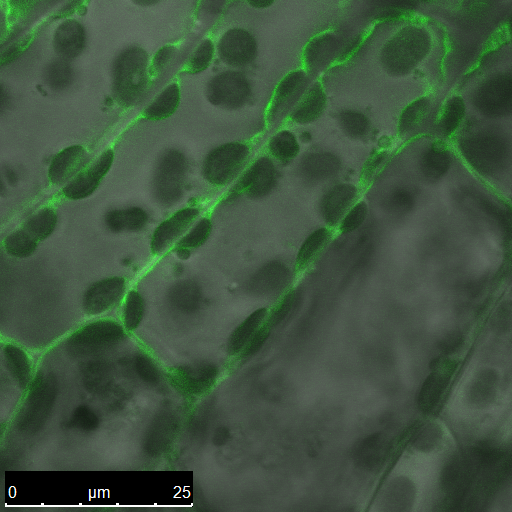

Supplement: S3 File — (ZIP) [file pone.0243620.s003.zip › 325-Venus/Experiment_Image199.tif]

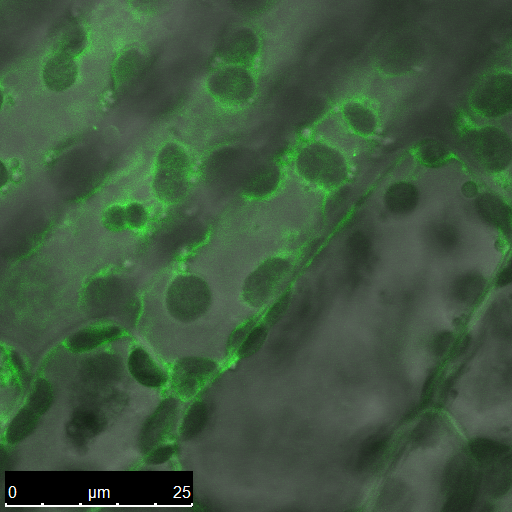

Supplement: S3 File — (ZIP) [file pone.0243620.s003.zip › 325-Venus/Experiment_Image201.tif]

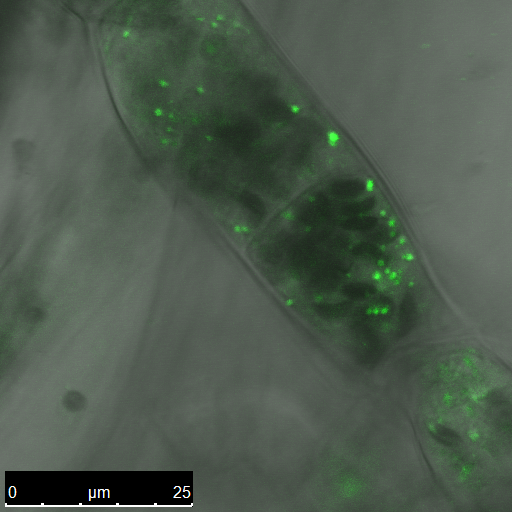

Supplement: S3 File — (ZIP) [file pone.0243620.s003.zip › 325-Venus/Experiment_Image203.tif]

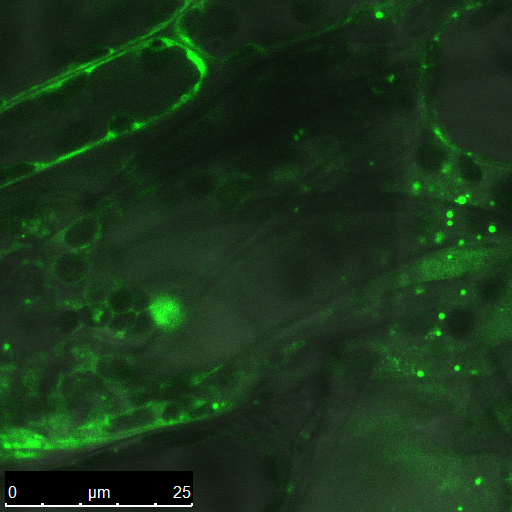

Supplement: S3 File — (ZIP) [file pone.0243620.s003.zip › 325-Venus/Experiment_Image205.tif]

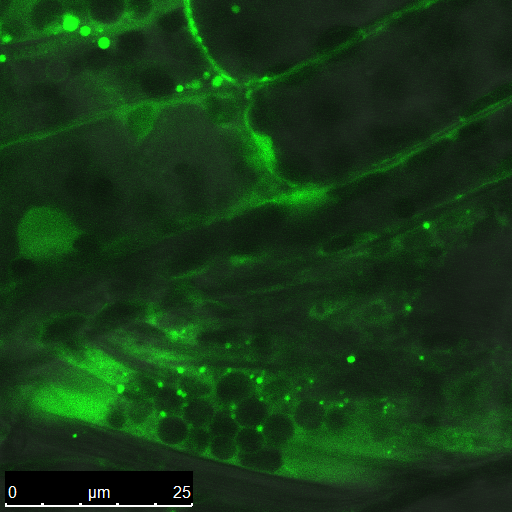

Supplement: S3 File — (ZIP) [file pone.0243620.s003.zip › 325-Venus/Experiment_Image207.tif]

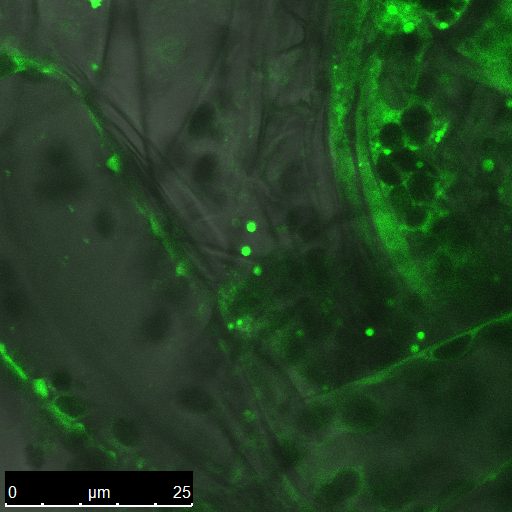

Supplement: S3 File — (ZIP) [file pone.0243620.s003.zip › 325-Venus/Experiment_Image209 325V end.tif]

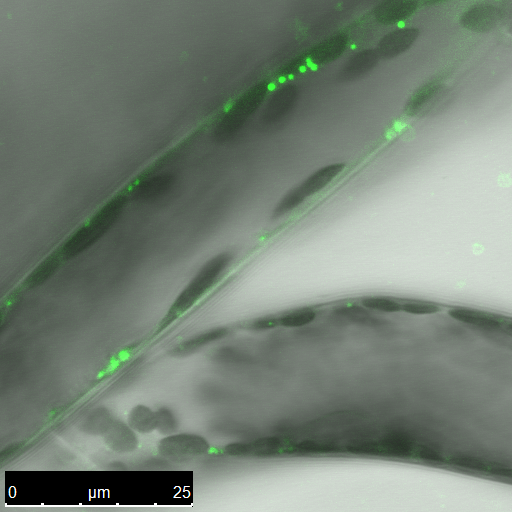

Supplement: S3 File — (ZIP) [file pone.0243620.s003.zip › Ole-LP42A-PTS/Experiment_Image238 ole lp42a pts begin.tif]

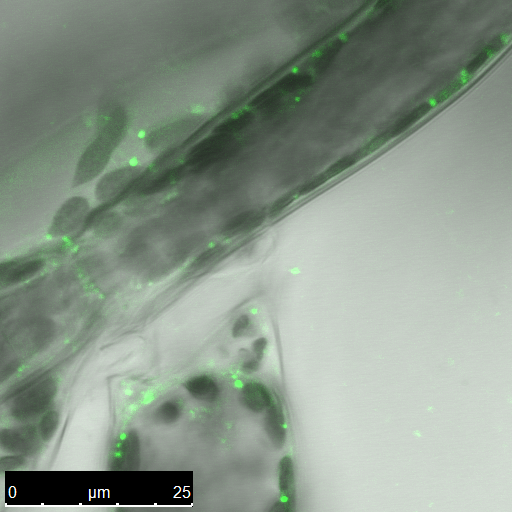

Supplement: S3 File — (ZIP) [file pone.0243620.s003.zip › Ole-LP42A-PTS/Experiment_Image240.tif]

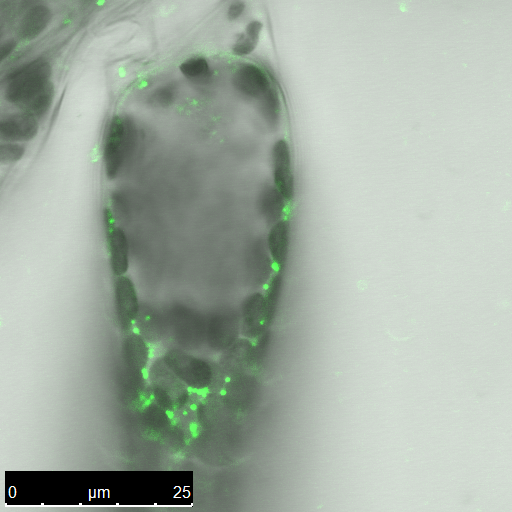

Supplement: S3 File — (ZIP) [file pone.0243620.s003.zip › Ole-LP42A-PTS/Experiment_Image242.tif]

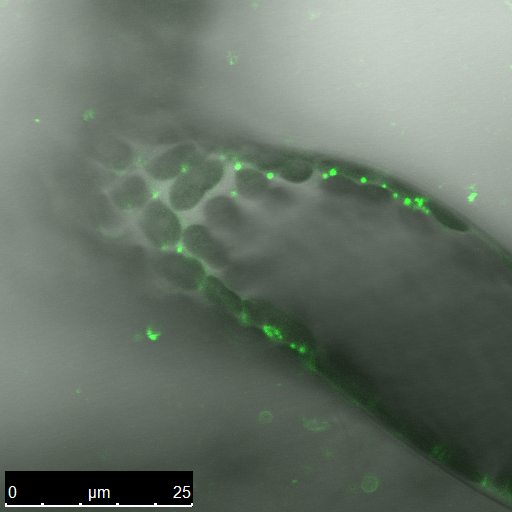

Supplement: S3 File — (ZIP) [file pone.0243620.s003.zip › Ole-LP42A-PTS/Experiment_Image244.tif]

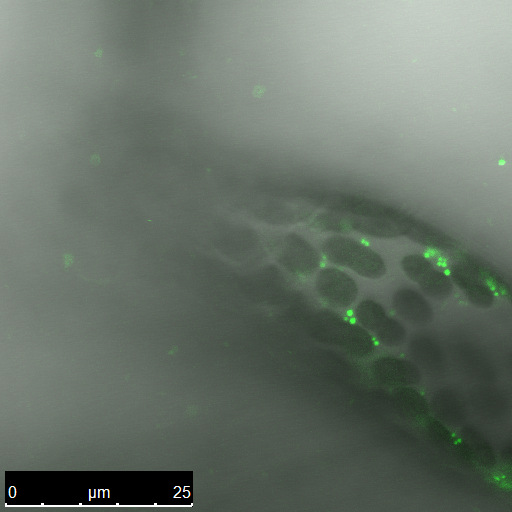

Supplement: S3 File — (ZIP) [file pone.0243620.s003.zip › Ole-LP42A-PTS/Experiment_Image246.tif]

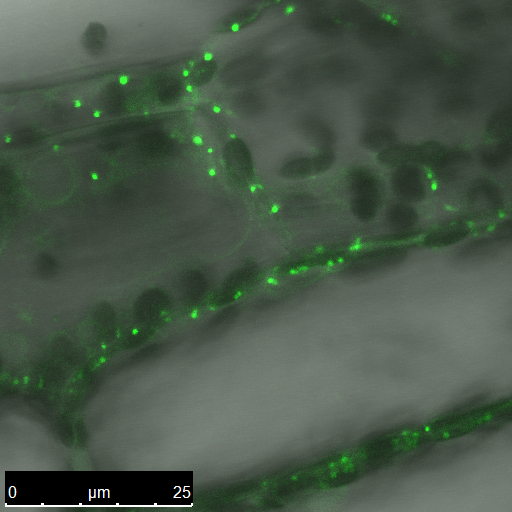

Supplement: S3 File — (ZIP) [file pone.0243620.s003.zip › Ole-LP42A-PTS/Experiment_Image248.tif]

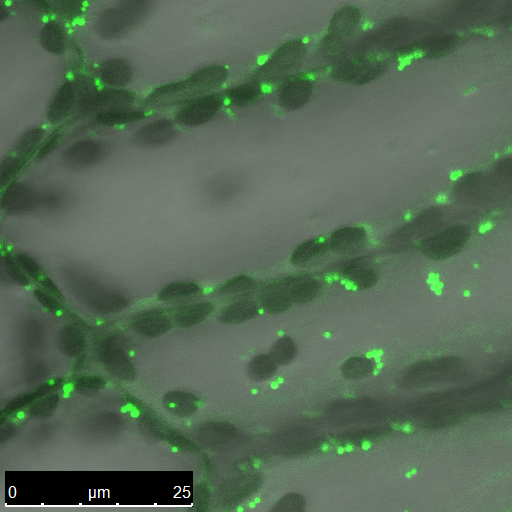

Supplement: S3 File — (ZIP) [file pone.0243620.s003.zip › Ole-LP42A-PTS/Experiment_Image250.tif]

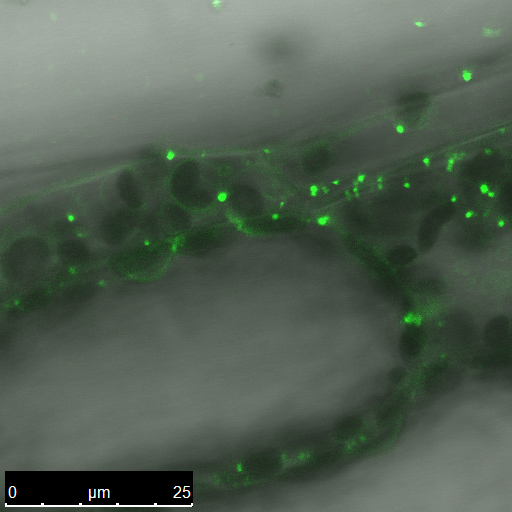

Supplement: S3 File — (ZIP) [file pone.0243620.s003.zip › Ole-LP42A-PTS/Experiment_Image252.tif]

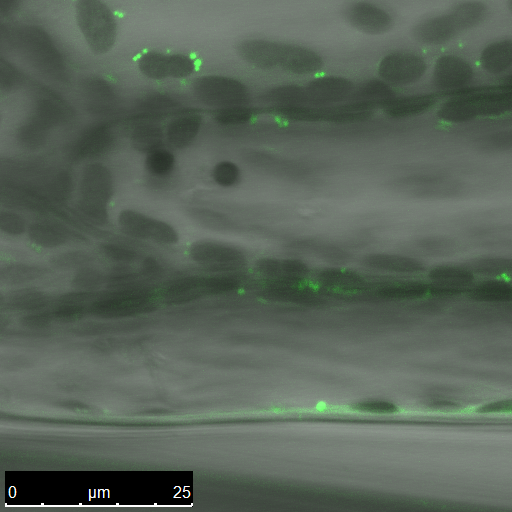

Supplement: S3 File — (ZIP) [file pone.0243620.s003.zip › Ole-LP42A-PTS/Experiment_Image254.tif]

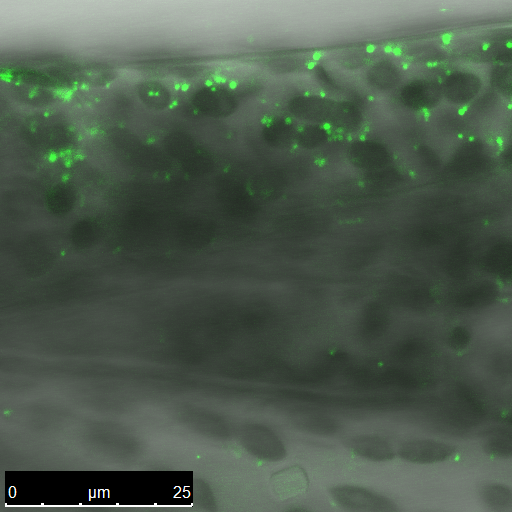

Supplement: S3 File — (ZIP) [file pone.0243620.s003.zip › Ole-LP42A-PTS/Experiment_Image256.tif]

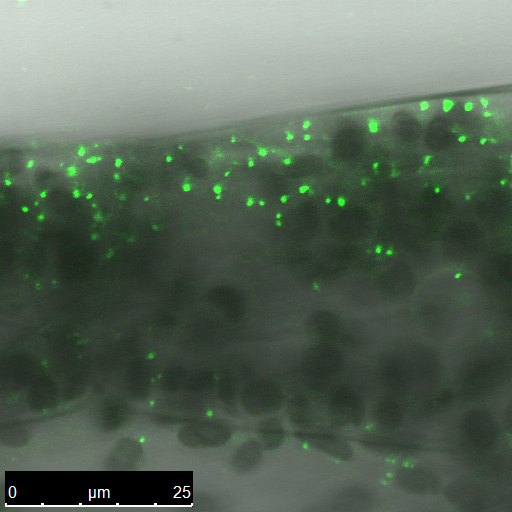

Supplement: S3 File — (ZIP) [file pone.0243620.s003.zip › Ole-LP42A-PTS/Experiment_Image258.tif]

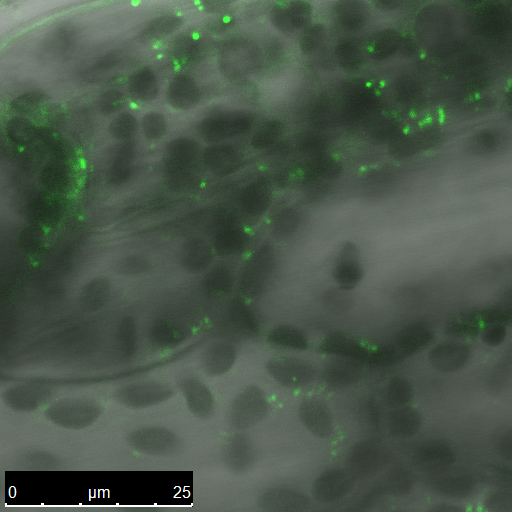

Supplement: S3 File — (ZIP) [file pone.0243620.s003.zip › Ole-LP42A-PTS/Experiment_Image260.tif]

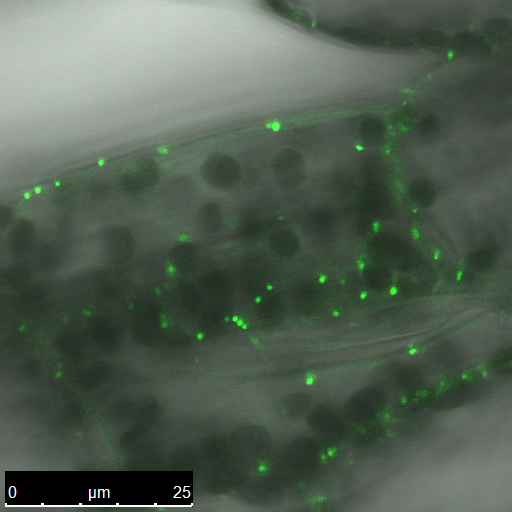

Supplement: S3 File — (ZIP) [file pone.0243620.s003.zip › Ole-LP42A-PTS/Experiment_ole lp4 2a pts end image 260.tif]

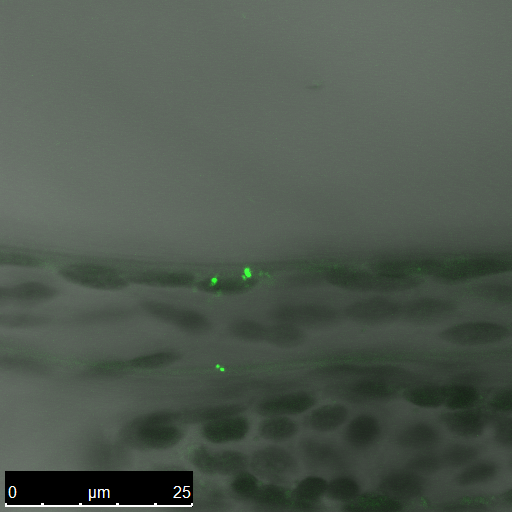

Supplement: S3 File — (ZIP) [file pone.0243620.s003.zip › PTS only/Experiment_Image151.tif]

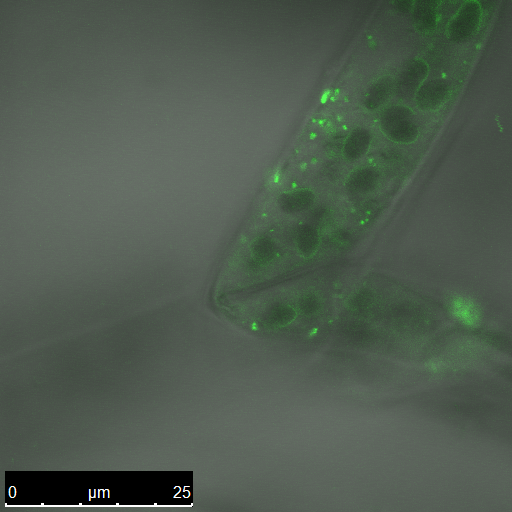

Supplement: S3 File — (ZIP) [file pone.0243620.s003.zip › PTS only/Experiment_Image153.tif]

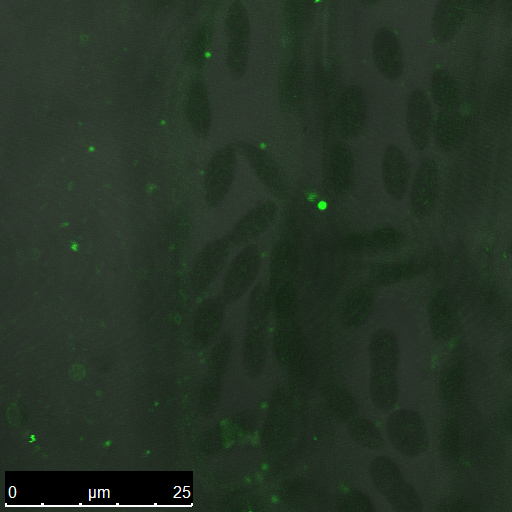

Supplement: S3 File — (ZIP) [file pone.0243620.s003.zip › PTS only/Experiment_Image156.tif]

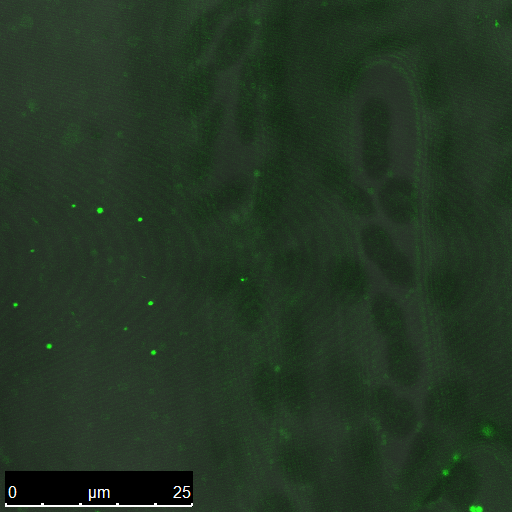

Supplement: S3 File — (ZIP) [file pone.0243620.s003.zip › PTS only/Experiment_Image158.tif]

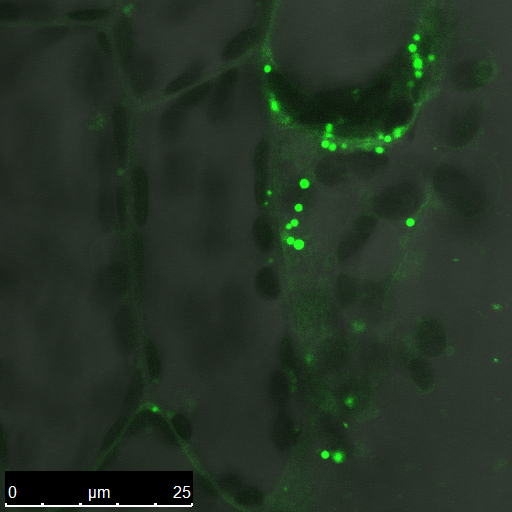

Supplement: S3 File — (ZIP) [file pone.0243620.s003.zip › PTS only/Experiment_Image160.tif]

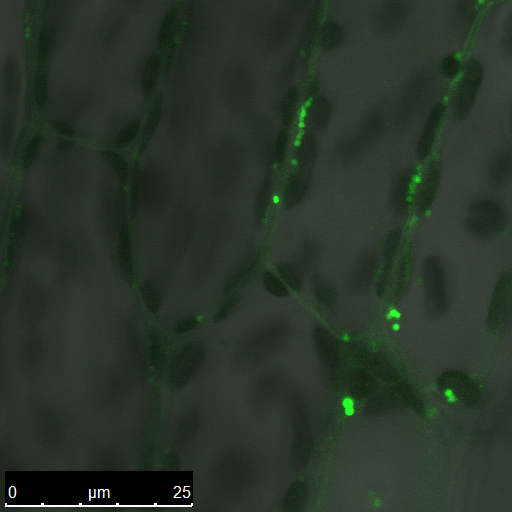

Supplement: S3 File — (ZIP) [file pone.0243620.s003.zip › PTS only/Experiment_Image162.tif]

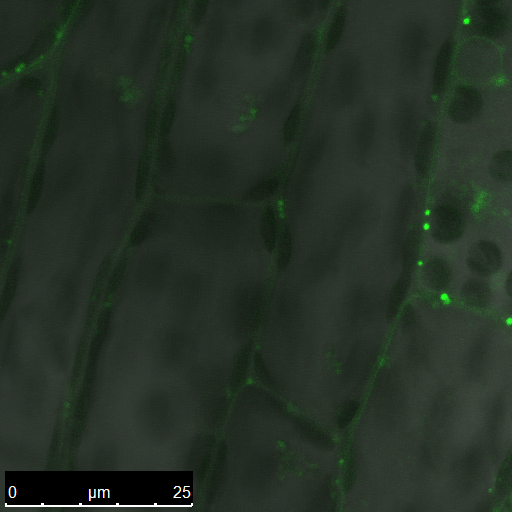

Supplement: S3 File — (ZIP) [file pone.0243620.s003.zip › PTS only/Experiment_Image164.tif]

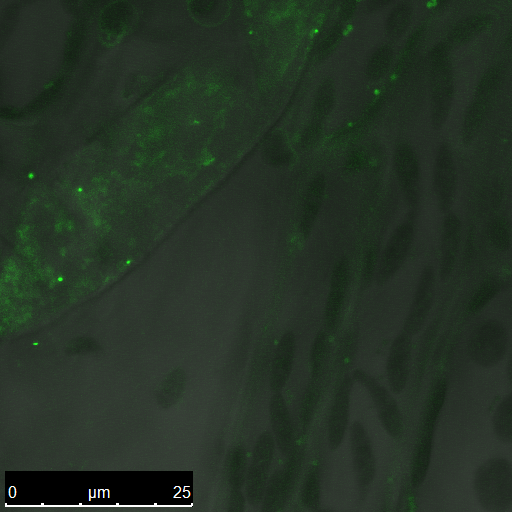

Supplement: S3 File — (ZIP) [file pone.0243620.s003.zip › PTS only/Experiment_Image166.tif]

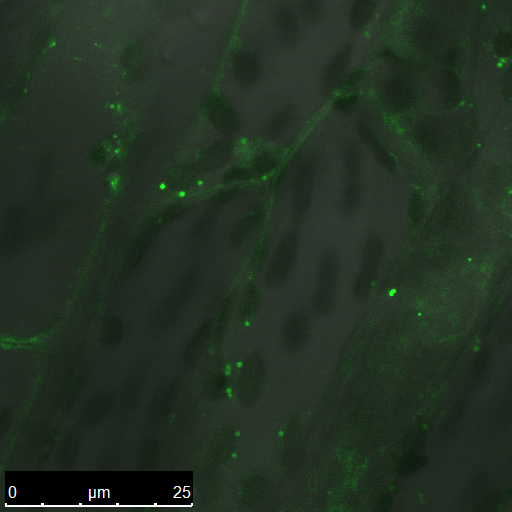

Supplement: S3 File — (ZIP) [file pone.0243620.s003.zip › PTS only/Experiment_Image168.tif]

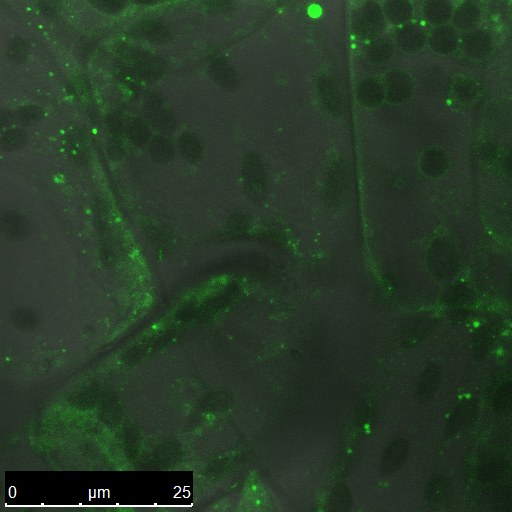

Supplement: S3 File — (ZIP) [file pone.0243620.s003.zip › PTS only/Experiment_Image170.tif]

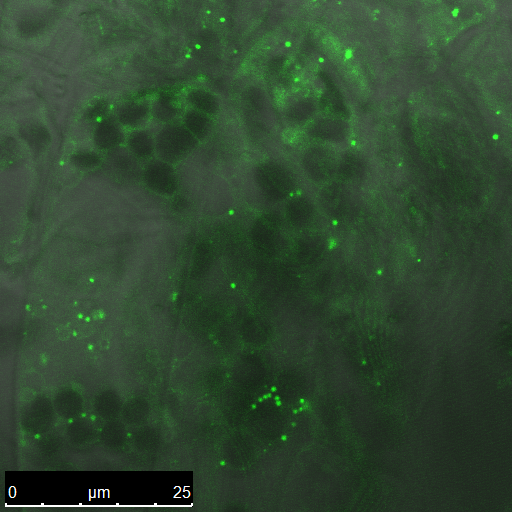

Supplement: S3 File — (ZIP) [file pone.0243620.s003.zip › PTS only/Experiment_Image172.tif]

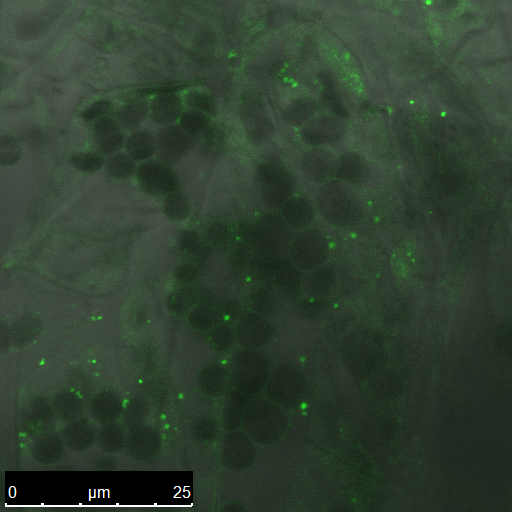

Supplement: S3 File — (ZIP) [file pone.0243620.s003.zip › PTS only/Experiment_Image174.tif]

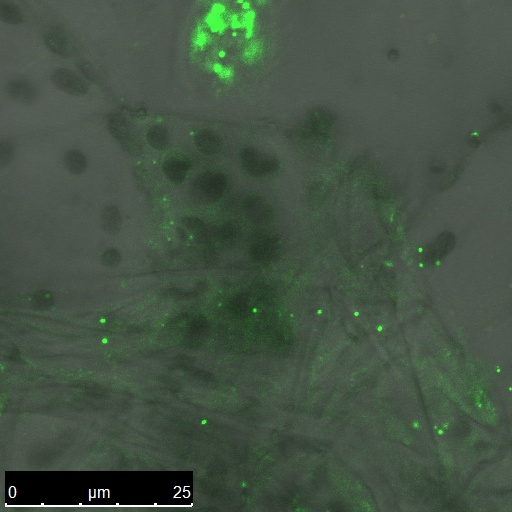

Supplement: S3 File — (ZIP) [file pone.0243620.s003.zip › PTS only/Experiment_Image176.tif]

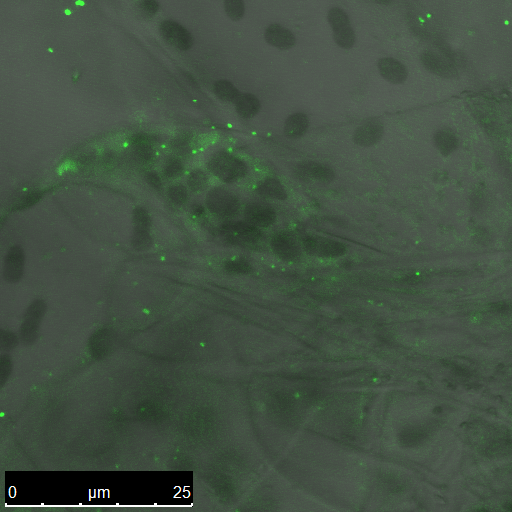

Supplement: S3 File — (ZIP) [file pone.0243620.s003.zip › PTS only/Experiment_Image178.tif]

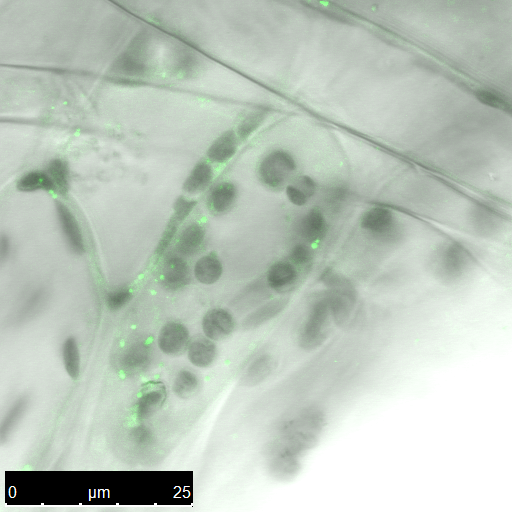

Supplement: S3 File — (ZIP) [file pone.0243620.s003.zip › PTS only/Experiment_PTS only begining 149.tif]

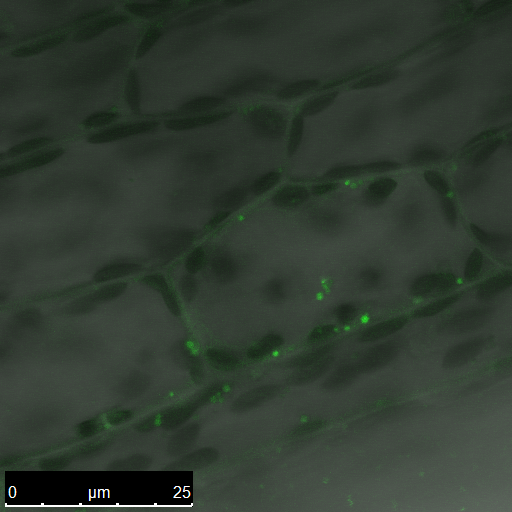

Supplement: S3 File — (ZIP) [file pone.0243620.s003.zip › PTS only/Experiment_PTS only end 180.tif]

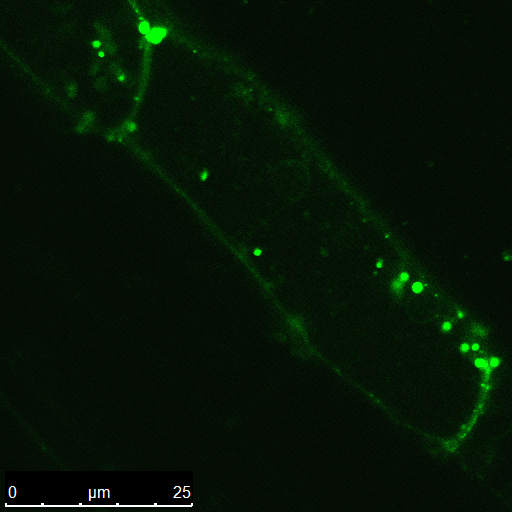

Supplement: S3 File — (ZIP) [file pone.0243620.s003.zip › WT/Experiment_wt 10.tif]

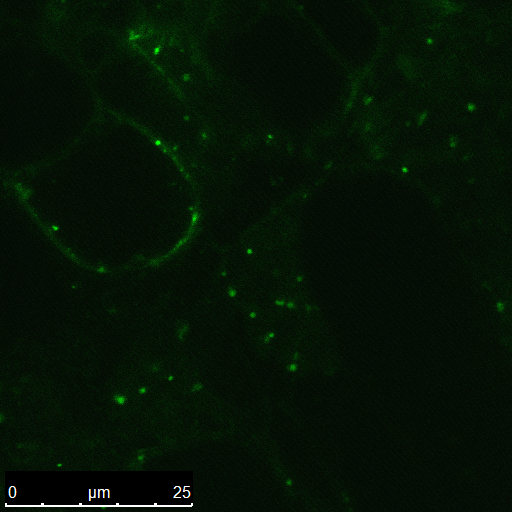

Supplement: S3 File — (ZIP) [file pone.0243620.s003.zip › WT/Experiment_wt 11.tif]

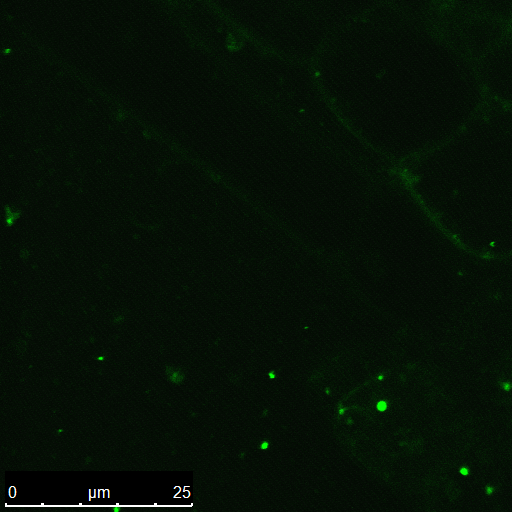

Supplement: S3 File — (ZIP) [file pone.0243620.s003.zip › WT/Experiment_wt 12.tif]

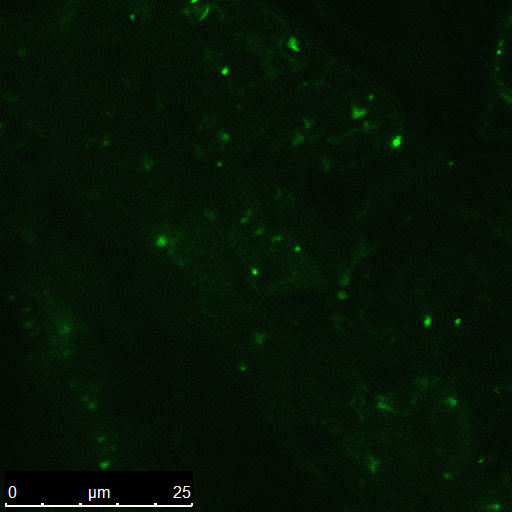

Supplement: S3 File — (ZIP) [file pone.0243620.s003.zip › WT/Experiment_wt 13.tif]

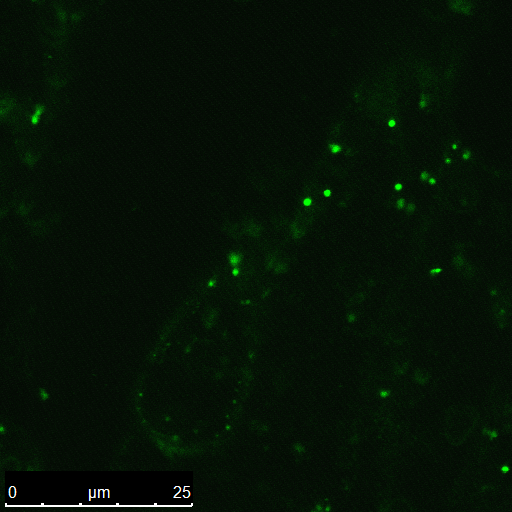

Supplement: S3 File — (ZIP) [file pone.0243620.s003.zip › WT/Experiment_wt 14.tif]

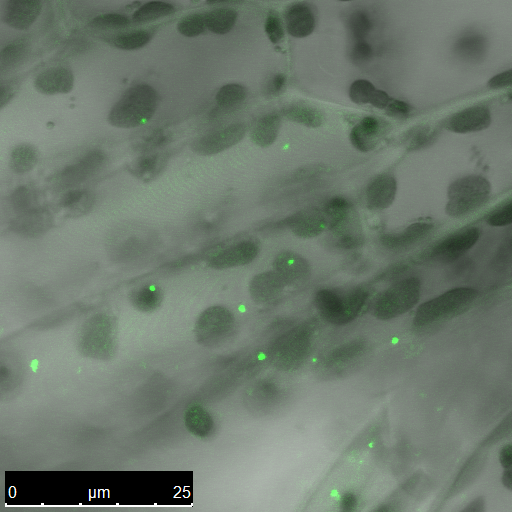

Supplement: S3 File — (ZIP) [file pone.0243620.s003.zip › WT/Experiment_wt 2.tif]

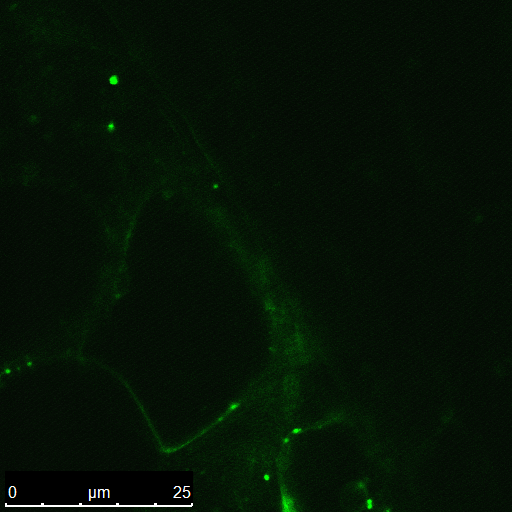

Supplement: S3 File — (ZIP) [file pone.0243620.s003.zip › WT/Experiment_wt 3.tif]

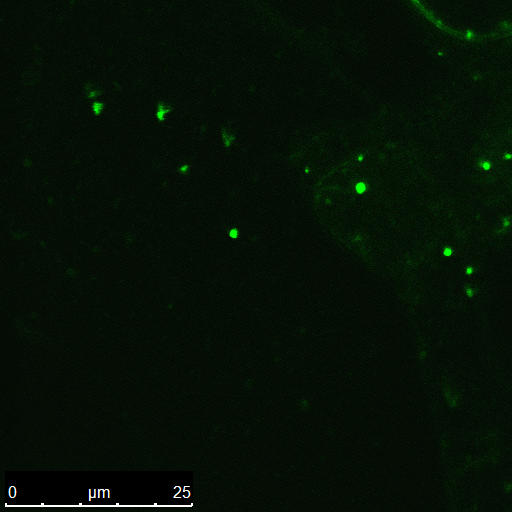

Supplement: S3 File — (ZIP) [file pone.0243620.s003.zip › WT/Experiment_wt 4.tif]

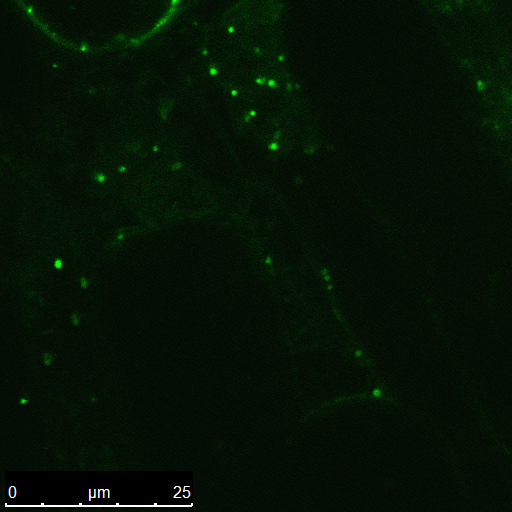

Supplement: S3 File — (ZIP) [file pone.0243620.s003.zip › WT/Experiment_wt 5.tif]

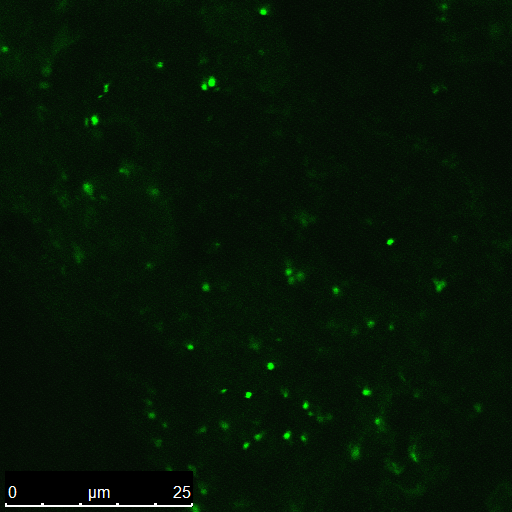

Supplement: S3 File — (ZIP) [file pone.0243620.s003.zip › WT/Experiment_wt 6.tif]

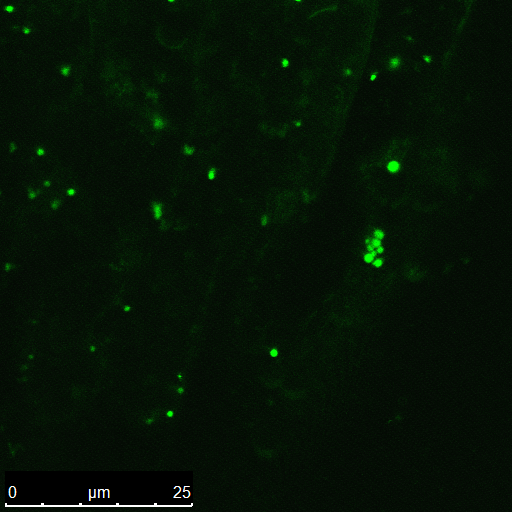

Supplement: S3 File — (ZIP) [file pone.0243620.s003.zip › WT/Experiment_wt 7.tif]

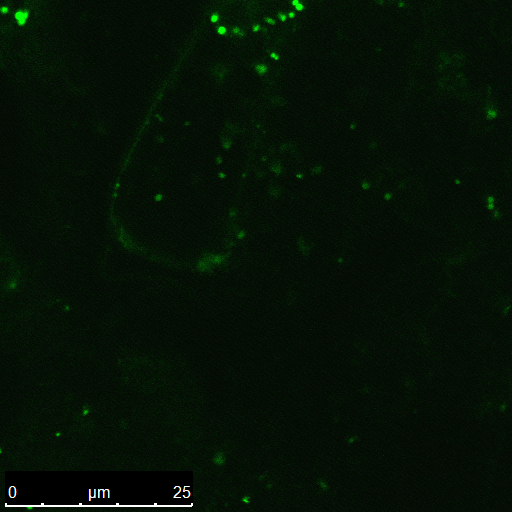

Supplement: S3 File — (ZIP) [file pone.0243620.s003.zip › WT/Experiment_wt 8.tif]

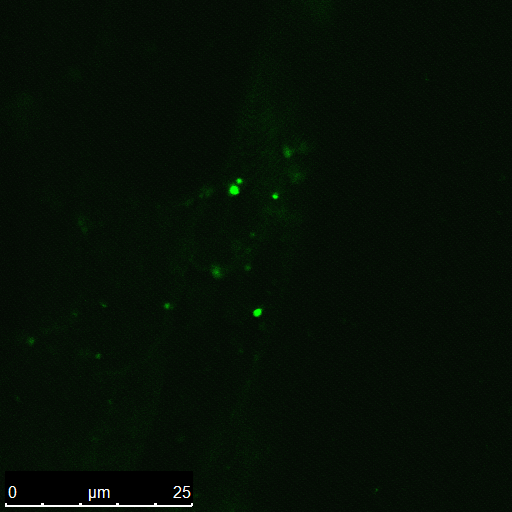

Supplement: S3 File — (ZIP) [file pone.0243620.s003.zip › WT/Experiment_wt 9.tif]
